# Supplementary material for: The topological requirements for robust perfect adaptation in networks of any size
Source: Nat Commun. 2018 May 1;9:1757. doi: 10.1038/s41467-018-04151-6 (PMC5931626; doi:10.1038/s41467-018-04151-6)
Supplement: Supplementary file 1 — Supplementary Information [file 41467_2018_4151_MOESM1_ESM.pdf]

Supplementary Information

Araujo and Liotta. The topological requirements for robust perfect adaptation in networks of any size.

Supplementary Note 1: Definitions

ROBUST PERFECT ADAPTATION (RPA): In this work, the term *perfect adaptation* is understood to describe a response of a "connected and transmissive" network (as defined below) in which a designated output node always returns to the same steady state (its "set point", or reference/baseline level) regardless of the level of stimulus delivered to a designated *input node*. Moreover, '*robust* perfect adaptation' (RPA) is taken to describe perfect adaptation that is (a) not dependent on particular parameter choices (cf. *tunable* perfect adaptation), and (b) is able to be implemented over a wide range of stimulus strengths, failing only when abundances of one or more network components become limiting. We also consider that the perfectly-adaptive steady-state of the output node should not be limited to trivial steady-states alone, eg. the steady-state activation necessarily returns to zero in response to a change in stimulus at the input node. We comment on the notion of RPA in the special case of trivial steady states only (eg. "state-dependent inactivation", as identified by [8]) in Supplementary Note 6.

DEFINITION OF A NETWORK FOR THE RPA PROBLEM: We consider a network to be formed by the interactions of collection of $p \in \mathbb{Z}^+$ interacting molecules (eg. proteins, RNA transcripts, genes, or a combination of these entities). These nature of these interactions will give rise to a number (say, $n \in \mathbb{Z}^+$) of entities which have the status of nodes.

A node is taken to be any entity that can encode and/or transmit a biochemical signal; most commonly a node corresponds to a molecule (its concentration or a particular activation state), a complex of molecules, or even a mathematical function of multiple biomolecular entities (see example discussed in Supplementary Note 5 (Supplementary Figure 17) for instance). Moreover, a node has the distinguishing feature of undergoing changes in its activity due to the influences of other nodes in the network. The concept of activity is to be understood in a very general sense here: in many contexts, protein activity may be governed by the status of its post-translational modifications; in contexts such as transcription networks or other gene regulatory networks, on the other hand, activity could simply refer to the expression level (abundance/concentration) of the molecule. Protein nodes, in particular, may assume the form of covalent modification cycles where the node undergoes interconversion among multiple activation states (commonly two, but sometimes more) via enzyme-catalysed reactions. In these cases, the node itself is represented by the active form of the protein; if more than one active form exists, all activation states are to be considered separate nodes. In cases where a node represents a mathematical function of certain signaling molecules (a ratio of pro- and anti-antiapoptotic factors, say, or a product of molecular concentrations (see Supplementary Note 5), the activity of the node is simply the numerical value of the quantity in question.

The nodes of a network thus form the essential structure or 'backbone' of the network and are the only quantities that require explicit consideration as variables in the mathematical formulation of the RPA problem. The remaining "non-nodal" network components play other ancillary roles in the transmission of biochemical signal through the network, and could include such elements as scaffolds, adaptor proteins, second messengers, say; the presence of such molecules need only be considered implicitly via their contribution to network parameters.

Moreover, in the study of robust perfect adaptation, we are interested in the question of how a particular node responds to the influence of a perturbation at some other specific location in the network. For this reason, of the n network nodes $P_1, \ldots, P_n$, two nodes are accorded a special status: (1) an input node, which is subjected to a signal or perturbation from outside the network, and (2) an output node, which is the 'endpoint' of interest. The input node and output node may be separate nodes, but can in principle be one and the same node. In the context of RPA, a network is considered 'robustly perfectly adaptive', or an RPA network, if and only if the output node always returns to the same activity level following any persistent alteration to the incoming signal received at the input node. While in many RPA networks, nodes other than the output node may also exhibit the RPA property (ie a perfectly adaptive response), only the output node is required to exhibit the RPA property in order for the network as a whole to be considered an RPA network. We emphasise also that RPA is a qualitative behaviour and independent of the magnitude of the steady-states of any of the network nodes.

CONNECTED AND TRANSMISSIVE NETWORKS: To play a role in RPA, a network node must be both *connected* and *transmissive* according to the following definitions.

Any node other than the input/output node(s) is connected if it influences the activation/deactivation rate of at least one other node in the network and is itself influenced by at least one other node. An input node is connected if its activation/deactivation rate is influenced by some signal or perturbation from outside the network (the 'input', I) and itself influences the activation/deactivation of one or more other nodes. An output node is connected if its activation/deactivation is influenced by at least one other node. Optionally, each of the n nodes may also influence their own activation/deactivation ('autoregulation'), the input node may also be activated/deactivated by one or more other nodes, and the output node may also activate/deactivate one or more other nodes.

Moreover, a node is considered transmissive if there exists at least one direct or indirect chain of node-node interactions linking the node to the input node, and at least one direct or indirect chain of node-node interactions linking it to the output node.

Thus, for the purposes of the RPA problem, a network is restricted to those nodes that are both connected and transmissive for a given choice of input and output nodes, permitting a mathematical analysis of the transmission of biochemical signal from input to output in response to alterations in the system input.

TIMESCALE OF RPA AND NETWORK FIXITY: We consider the RPA problem in the context of a network that has the property of *fixity* on the timescale of adaptation. That is, nodes are not created or destroyed on the timescale of adaptation, nor are interconnections between the nodes or their fundamental nature (ie. the characteristic of being activating or inhibitory) created or destroyed on this timescale.

REACTION-KINETIC AXIOMS: In the interests of generality, we wish to make no *a priori* assumptions as to the qualitative or quantitative aspects of chemical reaction rates (and their dependencies on the activities/abundances of individual reactants) involved in the transmission of biochemical signal among nodes. In particular, we do not appeal to any established reaction law or kinetic model, such as Mass Action kinetics, Michaelis-Menten kinetics, or cooperative kinetics. Indeed, it is one of the objectives of this work to identify the constraints on reaction kinetics that are required for a network to exhibit RPA.

On the other hand, we do insist that all network reactions conform to the following general properties of biomolecular interactions and enzyme-catalysed reactions:

1. The activities (or abundances) of all nodes must always be non-negative;

2. The input signal (or perturbation) to the network, I, may be either positive or negative, depending on the nature of the input. Positive values reflect an activating influence on the input node, while negative values correspond to an inhibitory influence;

3. Under conditions of network fixity (see definition in the previous section), the influence of a node on any particular reaction in the network must be either always activating in nature, or always inhibitory in nature, or zero (no influence). In other words, a node cannot alternate between activating and inhibitory roles in a particular reaction for different regions of the network's state space;

4. The reaction rate of each node should be a smooth function of the activities of each of the reactant nodes;

5. In studying the problem of RPA in generality, we restrict attention to networks that have the ability, at least in principle, to possess a non-trivial steady-state. This could be achieved, for instance, by reactions comprising two independent opposing reactions (eg. in a covalent modification cycle such as phosphorylation/dephosphorylation, or in the synthesis/degradation of a gene product.)

Supplementary Note 2: The Mathematical Theory of Robust Perfect Adaptation (RPA)

SN2.1 Mathematical Basis of RPA

Although 'asymptotic tracking' problems (of which the RPA problem is a special case) have been extensively studied in engineering control systems [9, 10, 21], we wish to develop a mathematical framework for which topological information may readily be established for the self-organising, self-regulating, evolvable RPA networks that occur in biology.

To this end, we consider a signaling network comprising n nodes, $P_1, P_2, \ldots, P_n$, as defined in Supplementary Note 1, with a designated input node, P_I, $I \in \{1, \ldots, n\}$, which receives the input, $\mathscr{I}$, to the network. The activity of such a network may be encoded by a set of n equations with general forms:

$$\begin{aligned} \frac{dP_I}{dt} &= f_I(P_1, \ldots, P_n, \mathscr{I}) \\ \frac{dP_i}{dt} &= f_i(P_1, \ldots, P_n), \quad i \in \{1, \ldots, n\}, i \neq I, \end{aligned} \tag{1}$$

where $f_I : \mathbb{R}^{n+1} \to \mathbb{R}$ and $f_i : \mathbb{R}^n \to \mathbb{R}$ denote the net activation rates at the respective nodes.

The differential of $\mathbf{f} = [f_1, \ldots, f_n]^T$ is then

$$\mathbf{df} = J_n \mathbf{dP} + \frac{\partial f_I}{\partial \mathscr{I}} \mathbf{e_I} d\mathscr{I}, \tag{2}$$

where $\mathbf{df} = [df_1, \ldots, df_n]^T$, $\mathbf{dP} = [dP_1, \ldots, dP_n]^T$, $J_n = \frac{\partial(f_1, \ldots, f_n)}{\partial(P_1, \ldots, P_n)}$ is the $n \times n$ Jacobian matrix of $\mathbf{f}$, and $\mathbf{e_I}$ is the unit vector along the coordinate axis associated with the variable P_I (ie. the designated input node).

For a given value of $\mathscr{I}$, a steady-state of the network, assuming at least one exists, will be denoted by $\pi_n = \{P_1^*, \ldots, P_n^*\}$. Hereafter, a superscript asterisk always denotes the steady-state activity of the associated node. Viewing $\mathscr{I}$ as a continuous parameter, the network steady-states defined by $\mathbf{f} = \mathbf{0}$ form a one-dimensional manifold embedded in $\mathbb{R}^n$ – a steady-state trajectory. In general, multiple steady-states could exist for some or all values of $\mathscr{I}$. In Supplementary Note 8, we note the conditions for the topological conjugacy of the linearized network to the corresponding non-linear network.

The fundamental question we address in the present work may now be expressed as follows: what are the characteristics of any n-node network, topologically and chemically (in terms of reaction-kinetics), that constrain that network to a steady-state trajectory that is embedded entirely within an $(n-1)$-dimensional affine subspace of $\mathbb{R}^n$, defined by $P_O^* = K$, where P_O^* is the steady-state activity of the designated output node, and K is some 'constant' that depends only on network parameters (kinetic rate constants, say). Now, from Equation (2), the steady-state trajectory satisfies

$$\begin{aligned} \frac{d\mathbf{P}^*}{d\mathscr{I}} &= -J_n^{-1*} \frac{\partial f_I^*}{\partial \mathscr{I}} \mathbf{e_I} \\ &= -\frac{\mathrm{adj}(J_n^*)}{\det(J_n^*)} \frac{\partial f_I^*}{\partial \mathscr{I}} \mathbf{e_I}. \end{aligned}$$

For the purposes of clarity, the superscript asterixes will hereafter be omitted from the Jacobian determinants and their component partial derivatives. From here onwards, these terms will always be taken to be evaluated at the network's steady-state, unless specified otherwise.

Then, the sensitivity of the designated output node (P_O)'s steady-state to the input is expressed by

$$\frac{dP_O}{d\mathscr{I}} = -\frac{\partial f_I}{\partial \mathscr{I}} \frac{\det(M_{IO})}{\det(J_n)}, \tag{3}$$

where M_{IO} is the $(n-1) \times (n-1)$ '*input-output minor*' of J_n, ie. the matrix obtained by removing the 'input row' and the 'output column' from J_n.

Now, robust perfect adaptation of the network, as defined earlier, requires $\frac{dP_O}{d\mathscr{I}} = 0$ **for all** $\mathscr{I}$ **and all** π_n. Therefore, RPA is implemented exactly when $\det(M_{IO})/\det(J_n) = 0$, which requires

$$\det(M_{IO}) = 0, \quad \text{and} \tag{4}$$

$$\det(J_n) \neq 0, \tag{5}$$

for all $\mathscr{I}$ and all π_n. Hereafter, we refer to the condition (4) as *the RPA equation*, and (5) as *the RPA constraint*.

We note in passing here that Equation (3) suggests a special case of RPA that occurs when $\frac{\partial f_I}{\partial \mathscr{I}} = 0$. We consider this special case in Supplementary Note 6.

We also observe that the RPA equation is a potentially huge equation in general, comprising some subset of the $(n-1)!$ terms corresponding to a "fully-connected" network of n nodes. A fifteen node network, for instance, results in an RPA equation of up to 8.7 x 10^{10} terms. Doubling the network size to thirty nodes produces an RPA equation of up to 8.8 x 10^{30} terms (see Supplementary Table 1 below).

Hence, our goal is to identify the set of all topological and chemical/reaction-kinetic constraints (ie. the set of arguments, and the functional form, for each f_k ($k \in \{1,\ldots,n\}$) in Equations (1)) imposed by (4) and (5) together.

SN2.2 Mathematical Form of the Reaction-Kinetic Axioms

The set of reaction-kinetic axioms outlined in Supplementary Note 1 may now be restated in mathematical terms:

1. $P_k \in \mathbb{R}^+$ for all $k \in \{1,\ldots,n\}$;
2. $\mathscr{I} \in \mathbb{R}$;
3. Each reaction $f_k(P_1,\ldots,P_n)$ must be a monotone function with respect to each of its arguments P_m individually. That is, $\frac{\partial f_k}{\partial P_m}$ must be either always non-negative or always non-positive for any $k, m \in \{1,\ldots,n\}$; it cannot change sign;
4. ∇f_k (for all $k \in \{1,\ldots,n\}$) must exist for all $\mathscr{I}$;
5. Each f_k ($k \in \{1,\ldots,n\}$) must be expressible in a form $f_k = f_k^{(+)} - f_k^{(-)}$, where $f_k^{(+)}$ represents the activating/synthesizing contribution to the overall reaction and $f_k^{(-)}$ represents the deactivating/degrading contribution.

Supplementary Table 1: Network Complexity Grows Factorially with the Number of Network Nodes

**Number of Network Nodes,** n	**Maximum Number of Terms in the RPA Equation** $= (n-1)!$
3	2
4	6
6	120
10	362,880
15	8.7 x 10^{10}
20	1.2 x 10^{17}
30	8.8 x 10^{30}

SN2.3 Topological Interpretation of the RPA Equations

Before developing a mathematical solution to the RPA problem, it is expedient to make explicit the relationship of the mathematical terms $\det(J_n)$ and $\det(M_{IO})$, which appear in the RPA conditions (4) and (5), to the network's topology and reaction kinetics.

Consider that each factor in each term of J_n is an element of the form $\frac{\partial f_a}{\partial P_b}$, evaluated at the network's steady-state. When $a \neq b$, we can consider such an element to represent a *link*, since it reflects the sensitivity, at steady-state, of the reaction rate at node P_a to the activity of node P_b - in a sense, the 'strength' of the network interaction **from** node P_b **to** node P_a, ie. $P_b \to P_a$. Moreover, a positive value for such a quantity reflects an activating influence of P_b on P_a, while a negative value indicates an inhibitory influence. Quantities of this form, in encoding the strength and nature of the interactions among network nodes, are fundamentally topological quantities; together, they establish the interconnectedness of the network. When $\frac{\partial f_a}{\partial P_b} = 0$, either node P_b does not regulate the activity of node P_a, or the reaction is "saturated" with respect to P_b; either way, we consider the link $P_b \to P_a$ to be absent.

On the other hand, the terms residing on the Jacobian diagonal, where $a = b$, are not related to the interconnectivity of nodes and are thus not topological quantities. Rather, these terms reflect the sensitivity of a node's reaction kinetics at steady-state to the node's own activity, and are thus node-specific *reaction-kinetic* terms. We may consider these terms to be *1-node cycles* (or simply '1-cycles'). It follows from our stated requirement for individual nodes to be able to possess a non-trivial steady state that the numerical value of these 1-cycles will always be non-positive. Importantly, these reaction-kinetic quantities may be identically zero under certain circumstances (to be discussed in Supplementary Note 3) - a property that does not affect the topology of the network. In other words, these are the only factors within the terms of $\det(J_n)$ and $\det(M_{IO})$ that can assume a zero value without removing any links from the network. Because of this special property, it is convenient to give these quantities a special name - *kinetic multipliers* - for reasons that will become clear as the analysis proceeds.

Definition 1. *The kinetic multiplier for the node P_k , $k \in \{1, \ldots, n\}$, is the quantity $\frac{\partial f_k}{\partial P_k}$ evaluated at the network steady-state.*

We now employ these ideas to formulate a network interpretation of $\det(J_n)$. It follows from the properties of the determinant that each of the $n!$ terms in the expansion is a unique product of n links and 1-cycles (kinetic multipliers) in which each of the n network nodes appears exactly once as 'the node' and exactly once as 'the regulator'. Thus, from a topological point of view, each term represents a unique combination of network circuits - either a single network circuit comprising all n nodes (hereafter, an 'n-cycle'), or the product of m p_i-cycles ($\sum_{i=1}^{m} p_i = n$) in which each network node participates in exactly one cycle; the m cycles are thus disjoint. We further note that each such 'cycle product' is sign adjusted, being prefixed by either a positive or negative sign. Thus, $\det(J_n)$ represents the sum of all possible sign-adjusted cycle-products for an n-node network.

Now, we further note that $\det(M_{IO})$ is the cofactor of the element $\frac{\partial f_I}{\partial P_O}$ (the link from the output node to the input node, $P_O \to P_I$) in the expansion for $\det(J_n)$. It follows that $\det(M_{IO})$ may be extracted from $\det(J_n)$ by: (i) first eliminating every term in $\det(J_n)$ that do not contain $\frac{\partial f_I}{\partial P_O}$ among its factors, then (ii) for the terms that remain, canceling the common $\frac{\partial f_I}{\partial P_O}$ link. Since each term of $\det(J_n)$ is a product of disjoint network cycles, removing the link $P_O \to P_I$ from its circuit in corresponds to 'breaking open' the circuit to yield a 'route' through the network from P_I to P_O. Thus, each term in the expansion for $\det(M_{IO})$ comprises a particular route through the network from input node to output node, along with a cycle-product comprising all network nodes that are disjoint from that route. The term retains the sign prefix of the parent-term in the $\det(J_n)$ expansion; the full $\det(M_{IO})$ expansion thus comprises all $(n-1)!$ possible sign-adjusted route-cycle combinations for an $n-$node network.

For explanatory convenience in the analysis to follow, it is helpful to make explicit the distinction between cycles involving a single node (kinetic multipliers, which do not play a topological role in the solution to the RPA problem) and cycles involving multiple nodes (which do play a topological role in the problem).

Definition 2. *For an n-node network, a k-cycle in its RPA equation, for* $2 \le k \le n$ *is a* **circuit**.

We now consider the signs of the various terms of $\det(J_n)$ and $\det(M_{IO})$. For later explanatory convenience, we introduce the following definitions:

Definition 3. *The* **native sign** *is the sign which prefixes the term in the original determinant expansion.*

Definition 4. *In a particular concrete realisation of a network, the individual factors within each term will take on a sign that reflects whether the node-node interaction encoded by that factor is an activating one (positive sign) or an inhibitory one (negative sign). We refer to the product of all such signs, over all factors for the individual term in question, as the term's* **influence sign**.

Definition 5. *The* **net sign** *for each term of the RPA equation is the product of its native sign and its influence sign.*

At this point in our exposition, we note that the **native sign** is a reflection of the number of cycles present in that term. We make this notion precise by the following Proposition:

Proposition 1. *The native sign of each term of the RPA equation is given by* $(-1)^{z+n+1}$*, where n is the number of nodes in the network and z is the number of distinct cycles present in the term.*

Proof. First we consider the signs associated to each term in $\det J_n$, noting that, by definition

$$\det J_n = \Sigma(-1)^k \frac{\partial f_1}{\partial P_{b1}} \frac{\partial f_2}{\partial P_{b2}} \cdots \frac{\partial f_n}{\partial P_{bn}}, \tag{6}$$

where the denominator subscripts $b1, b2, \ldots, bn$ are equal to $1, 2, \ldots, n$, taken in some order - there being $n!$ such orderings and thus $n!$ such terms in the summation. The exponent k represents the number of pairwise interchanges of elements in order for these denominator subscripts to be placed in the order $1, 2, \ldots, n$.

Now, we note that a particular term produced by this ordering is $\frac{\partial f_1}{\partial P_1} \frac{\partial f_2}{\partial P_2} \cdots \frac{\partial f_n}{\partial P_n}$, which represents n 1-cycles (being the maximum number of cycles in a term for the determinant of J_n). We further observe that the pairwise interchange of denominator subscripts for any two component factors $\frac{\partial f_i}{\partial P_i} \frac{\partial f_j}{\partial P_j}$ of this term will effectively join the two associated 1-cycles into a single 2-cycle, $\frac{\partial f_i}{\partial P_j} \frac{\partial f_j}{\partial P_i}$.

Indeed, each factor is a component of a cycle. Interchanging two denominator subscripts from factors contributing to separate cycles joins the associated two separate cycles into a single one; by contrast, interchanging two denominator subscripts from factors in the same cycle separates that circuit into two individual cycles. For example, considering the 4-node cycle represented by $\frac{\partial f_2}{\partial P_1} \frac{\partial f_3}{\partial P_2} \frac{\partial f_4}{\partial P_3} \frac{\partial f_1}{\partial P_4}$, exchange of the denominator subscripts 2 and 3 yields $\frac{\partial f_2}{\partial P_1} \frac{\partial f_3}{\partial P_3} \frac{\partial f_4}{\partial P_2} \frac{\partial f_1}{\partial P_4}$, which represents a 1-cycle (node 3) and a 3-cycle (involving nodes 1, 2 and 4).

Thus, each interchange increases OR decreases the number of cycles contained in a term by exactly 1. Now, the term representing n 1-cycles (being the identity permutation of denominator subscripts) is necessarily prefixed by a positive sign. Thus, from the above argument, it is clear that if n is even, all terms containing an even total number of cycles will also be prefixed by a positive sign , and all terms with an odd total number of cycles will be prefixed by a negative sign. Similarly, if n is odd, all terms containing an odd number of cycles will be prefixed by a positive sign and all terms with an even number of cycles will be prefixed by a negative sign. Thus, for all n, the native sign of each term is given by $(-1)^{z+n}$, where z $(1 \le z \le n)$ is the number of distinct cycles present in the term.

Having established the native sign for any term of $\det J_n$, we observe that the terms of $\det M_{IO}$ may be obtained from the set of terms of $\det J_n$, and thus inherit their native signs from the parent terms in $\det J_n$. In particular, we recall here that $\det M_{IO}$ may be obtained from $\det J_n$ in the following manner: First, all terms that do not contain the factor $\frac{\partial f_I}{\partial P_O}$ are discarded from $\det J_n$. (For those terms that now remain - those that do contain $\frac{\partial f_I}{\partial P_O}$ - the native sign is as described above.) Second, the factor $\frac{\partial f_I}{\partial P_O}$ is cancelled from term, 'breaking open' the cycle to which $\frac{\partial f_I}{\partial P_O}$ contributes, and thereby forming a route from P_I to P_O. In

this way, the number of cycles in each term is reduced by exactly one. It follows that the native sign of each term of the RPA equation is given by $(-1)^{z+n+1}$. □

Corollary 1. *For a given network, if all cycles have a negative influence sign, then the net sign of each term of the RPA equation is determined solely by the influence sign of the* **route** *component of that term. That is, for a given network, terms with a negative route will have the opposite sign from terms with a positive route.*

We remark in closing that for any *particular* network, a route or a circuit will be absent from the expansions for $\det(M_{IO})$ and $\det(J_n)$ (and from the associated network) if at least one of its links is absent. Thus, in general, $\det(J_n)$ for a particular network will comprise η cycle-products, with $1 \le \eta \le n!$, and $det(M_{IO})$ will comprise μ route-cycle combinations, with $1 \le \mu \le (n-1)!$.

SN2.4 Networks where a Single Node Acts as Both Input Node and Output Node

We must consider also the possibility of a special case where the input node and the output node are one and the same node. Denoting this single node by P_I, we can examine the topological interpretation of $\det(M_{II})$. To this end, we note that $\det(M_{II})$ may be obtained from $\det(J_n)$ by (i) first, eliminating all terms that do not involve the kinetic multiplier $\frac{\partial f_I}{\partial P_I}$ in that expansion, then (ii) cancelling this kinetic multiplier $\frac{\partial f_I}{\partial P_I}$ from each remaining term. Taking account of the topological interpretations of terms described in the preceding section, along with the notions of sign-adjustment presented there, it follows that $\det(M_{II})$ represents the set of all (sign-adjusted) disjoint cycle combinations that do not involve the node P_I (the input/output node). There are thus no routes present in a network in which the input and output nodes are a single node; only circuits and kinetic multipliers exist in such a network.

Supplementary Note 3: General Solution to the RPA Problem and the Mechanisms of Robust Perfect Adaptation

SN3.1 General Principles

Our goal is to identify **all the possible network topologies** whose RPA equation (4) admits a solution, subject to the RPA constraint (5). In other words, we want to consider the RPA problem topologically, and in complete generality - for networks of any size (number of nodes), any complexity (interconnectivities among nodes) and any types of chemical reaction mechanisms governing the interactions among nodes.

As noted in Supplementary Note 2, a network with n nodes has an RPA equation with up to $(n-1)!$ terms, and an RPA constraint with up to $n!$ terms, both of which must be satisfied for all inputs $\mathscr{I} \in \mathbb{R}$ and all network steady-states, π_n. Thus, for a network comprising just ten nodes, the RPA equation could contain as many as 362,880 terms, with an RPA constraint of as many as 3,628,800 terms - an enormous, and consequently complex, equation to solve in full generality.

We proceed as follows: Let $\mathscr{R}$ be the set of all (signed) terms in the RPA equation for an n-node network. The central idea in arriving at a general solution to the RPA problem is the concept of a 'minimally adaptive' (MA)-subset, and the partition of $\mathscr{R}$ into a collection of disjoint MA-subsets which cover $\mathscr{R}$.

Definition 6. *A minimally adaptive subset of $\mathscr{R}$ (hereafter, an MA-subset) is a non-empty set that satisfies the following two conditions:*

1. *The sum of its constituent terms is identically zero for all $\mathscr{I}$ and all π_n, making it an 'adaptive' set.*
2. *The set cannot be further subdivided into smaller subsets whose elements sum to zero independently. In other words, the set cannot be expressed as the union of disjoint adaptive sets. It is thus a 'minimally' adaptive set, because all its terms are strictly required for it to be adaptive.*

From this definition, it is immediately apparent that there are two fundamentally different types of MA-subsets: S-sets and M-sets.

Definition 7. *An S-set is a singleton MA-subset. In an S-set, the single constituent term is identically zero for all $\mathscr{I}$ and all π_n.*

Definition 8. *An M-set is a multi-term MA-subset. In an M-set, constituent terms are strictly non-zero for all $\mathscr{I}$ and all π_n.*

Now, consider the conditions under which an arbitrary collection of terms selected from $\mathscr{R}$ has the potential to form an MA-subset. To this end, we note that to any such subset we can associate two equations:

1. A local adaptation equation (LAQ). The LAQ is formed by equating the sum of the terms to zero, such that all terms are required to solve the equation (thereby making it an MA-subset).
2. A complementary adaptation equation (CAQ). The CAQ is formed by equating the terms of the complement in $\mathscr{R}$ of the MA-subset to zero, *not necessarily minimally.*

Definition 9. *An internally valid MA-subset is one for which a solution exists to its LAQ for all $\mathscr{I}$ and all π_n.*

Definition 10. *A relatively valid MA-subset is one for which a solution exists to its CAQ for all $\mathscr{I}$ and all π_n.*

Definition 11. *An MA-subset that is both internally and relatively valid is a* **valid MA-subset**.

Where a valid MA-subset can be identified, the problem reduces to an analysis of its complement in $\mathscr{R}$ which is either (i) itself minimally adaptive, or (ii) can be further partitioned into multiple valid MA-subsets. A partition of $\mathscr{R}$ into simultaneously valid MA-subsets thus represents a solution to the RPA equation for the associated n-node network.

SN3.2 Internally Valid MA Subsets

We now begin our consideration of the circumstances under which an arbitrary subset of $\mathscr{R}$ may form an internally valid MA subset. We consider the creation of S-sets and M-sets separately in turn.

SN3.2.1 Internally Valid S-sets

We recall from Supplementary Note 2 that kinetic multipliers are the only factors within the elements of $\mathscr{R}$ (ie. the terms of the RPA equation (4)) that can assume a zero value without altering the topology of the associated network. Thus, an S-set is formed when an element of $\mathscr{R}$ contains a cycle product with at least one kinetic multiplier whose steady-state value is identically zero for all $\mathscr{I}$. That is, we require $\frac{\partial f_o}{\partial P_o} = 0$ for some node P_o; such a condition creates an S-set from each element of $\mathscr{R}$ in which P_o appears **neither** as a route-node **nor** as a circuit-node. We refer to this particular constraint on the reaction kinetics of node P_o hereafter as *opposer kinetics*, and to the node P_o as an *opposer node*.

Definition 12. *An opposer node is any node,* $P_o \in \{P_1, \ldots, P_n\}$ *for which* $\frac{\partial f_o}{\partial P_o} = 0$ *for all* $\mathscr{I}$ *and all* π_n.

Under what circumstances (in terms of network configuration) can a node $P_o \in \{P_1, \ldots, P_n\}$ act as an opposer node?

Theorem 1. *A node* P_o *can adopt the special 'opposer' kinetics,* $\frac{\partial f_o}{\partial P_o} = 0$, **only** *if it participates in a network circuit.*

Proof. For *every* term in $\det(J_n)$, every node appears *either* in a circuit *or* as a kinetic multiplier (never both). Therefore, if a node P_o does not act as a participant in at least one circuit in the network, then it must appear as a kinetic multiplier in *every* term in $\det(J_n)$. Under these circumstances, if $\frac{\partial f_o}{\partial P_o} = 0$, then $\det(J_n) = 0$, thus violating the RPA constraint (5). □

Thus, for a node to adopt reaction kinetics where $\frac{\partial f_o}{\partial P_o} = 0$ for all $\mathscr{I}$ at the network's steady-state, and thus act as an *opposer node*, it must contribute to at least one network circuit. An opposer node will *oppose* any route, at least partially, if it is disjoint from that route - that is, it will create an S-set for any instance of a route in which it appears as a kinetic multiplier in that route's cycle component.

SN3.2.2 Creation of S-sets in the Network

It is instructive at this point in the exposition to determine the topological characteristics of a node, or *set* of nodes, that can oppose a route *fully* - that is, such that all instances of the route are assigned to S-sets; no M-set in the partition contains an instance of that route. A consideration of this problem yields Theorems 2 and 3:

Theorem 2. *A* **single opposer node** *fully opposes a route if and only if the node:*
(a) is disjoint from that route, and
(b) participates only in circuits that are contiguous with the route.

Proof. A node, P_o, fully opposes a route exactly when it appears in the form $\frac{\partial f_o}{\partial P_o}$ in the cycle component of every instance of the route in the RPA equation. Now, appearing anywhere in the cycle component of the term necessarily requires P_o to be disjoint from the route (condition (a)). Moreover, we know from Theorem 1 that $\frac{\partial f_o}{\partial P_o}$ must participate in a circuit in order to adopt the opposer kinetics, $\frac{\partial f_o}{\partial P_o} = 0$, required for creating S-sets from all such terms. If P_o participates in at least one circuit that is disjoint from the route, then that circuit containing P_o must appear in the cycle component of at least one of the instances of that route; for that/these terms, $\frac{\partial f_o}{\partial P_o}$ cannot appear in the cycle component (ie. P_o, being a circuit node, cannot also appear as a kinetic multiplier in that/these instance(s)). All circuits containing P_o must therefore be contiguous with the route in order for P_o to appear as a kinetic multiplier in all instances of the route, and thereby fully oppose the route (condition (b)).

Conversely, suppose a node is disjoint from a route and all circuits in which it participates are contiguous with the route. Since it is disjoint from the route (condition (a)), it will be contained in the cycle component of all instances of that route in the RPA equation. Moreover, since all circuits in which it participates are contiguous with the route, such circuits cannot appear in the cycle component of any instance of the route. Thus, every instance of the route has a cycle component containing $\frac{\partial f_o}{\partial P_o}$. Thus P_o fully opposes the route. □

Now, we learn from Theorem 2 that no node can fully oppose a route by itself if it contributes to (at least) one circuit in the network that is disjoint from the route in question. Nevertheless, the logical possibility remains that a *collection* of nodes, each of which is unable to fully oppose a route by itself (by virtue of its participation in a disjoint circuit), can cooperate with the other members of the collection to fully oppose the route. With this possibility in mind, we define an "opposing set" as follows:

Definition 13. *An Opposing Set for a route is a set of opposer nodes which, together, are necessary and sufficient to fully oppose a route.*

We now consider the requirements for a collection of nodes, each of which participates in at least one disjoint circuit with respect to a particular route, to constitute an opposing set for that route. We first note that to any such collection of nodes we may associate a larger set of nodes (the "master set") comprising *all* members of *all* disjoint circuits (with respect to the route in question) in which the members of the proposed opposing set participate. The following theorem can now be stated and proved:

Theorem 3. *A set of nodes, $\{P_{o1},\ldots,P_{om}\}$, constitutes an opposing set for a particular route if and only if, for each $P_o \in \{P_{o1},\ldots,P_{om}\}$, (a) P_o is disjoint from the route, (b) the complement of P_o (in the master set) contains either a single circuit, or a collection of mutually disjoint circuits, containing* **all other** *members of the opposing set, and (c) for each disjoint circuit in which P_o participates, the complement of that disjoint circuit in the master set* **does not** *contain* **any** *circuit, or collection of mutually disjoint circuits, containing all other members of the opposing set.*

Proof. Suppose the set $\{P_{o1},\ldots,P_{om}\}$ is an opposing set for a particular route. From the definition of the opposing set, all m nodes are required to fully oppose the route; no proper subset of $\{P_{o1},\ldots,P_{om}\}$ is sufficient to oppose the set, and no node that is not in the set is required for the route to be fully opposed. First, this requires that each member of $\{P_{o1},\ldots,P_{om}\}$ be in the cycle component of each instance of the route in the RPA equation, which in turn requires each member to be disjoint from the route (property (a)).

In addition, for each P_o in $\{P_{o1},\ldots,P_{om}\}$ to be *required* (necessary) to fully oppose the route, there must exist at least one instance of the route in the RPA equation in which P_o is the *only* member of the opposing set to appear in the form of a kinetic multiplier (otherwise that node is not strictly required to fully oppose the route). This requires that the complement of P_o in the master set contain mutually disjoint circuits (or a single circuit) that contain all other members of the opposing set (property (b)).

Now, for the set $\{P_{o1},\ldots,P_{om}\}$ to be sufficient to oppose the route, every instance of the route in the RPA equation must have at least one of the members of $\{P_{o1},\ldots,P_{om}\}$ appearing in the form of a kinetic multiplier. (There cannot exist any instance of the route in which all members of P_o appear as a member of a circuit). This requires that, for every P_o, the complement in the master set of each of its disjoint circuits (with respect to the route) does not contain mutually disjoint circuits (or a single circuit) that contain all other members of the opposing set (property (c)).

Thus, each member of an opposing set has the properties (a), (b) and (c).

Conversely, suppose each member of a set of nodes $\{P_{o1},\ldots,P_{om}\}$ has all properties (a)-(c). Property (a) implies that, in each instance of the route in question, each member of $\{P_{o1},\ldots,P_{om}\}$ is present either as a kinetic multiplier or as a circuit, which in turn implies that each member of $\{P_{o1},\ldots,P_{om}\}$, taken individually, partially opposes the route. Now, condition (c) implies that, for each $P_o \in \{P_{o1},\ldots,P_{om}\}$, for every instance of the route in which P_o appears as a cycle, at least one *other* member of $\{P_{o1},\ldots,P_{om}\}$ must appear in the form of a kinetic multiplier. Thus, properties (a) and (c) together imply that the members of $\{P_{o1},\ldots,P_{om}\}$ are sufficient to fully oppose the route.

Property (b), on the other hand, implies that for each $P_o \in \{P_{o1}, \ldots, P_{om}\}$, there exists at least one instance of the route in which P_o is the only member of $\{P_{o1}, \ldots, P_{om}\}$ appearing in the form of a kinetic multiplier. Thus, each member of $\{P_{o1}, \ldots, P_{om}\}$ is required (necessary) to oppose the route.

The set of nodes $\{P_{o1}, \ldots, P_{om}\}$ is thus an opposing set for a route.

□

Now, it follows from the properties of the members of an opposing set (Theorem 3, properties (b) and (c)) , that the family of disjoint circuits defining the "master set" is connected (ie. each disjoint circuit must be contiguous with at least one other disjoint set in the family). In Supplementary Note 4 to follow, after considering the chemical reaction laws that are actually required to *create* opposer nodes, we shall elaborate further on the topological structures within a network (implied by Theorem 3) that correspond to the activities of an opposing set.

Although Theorem 2 highlights the topological conditions, in terms of participation in circuits and relationship to route, under which a single opposer node may *fully oppose* a route *by itself*, we observe that a single opposer node - participating in **no** disjoint circuits with respect to the route in question - vacuously satisfies the conditions of Theorem 3. Single opposer nodes are topologically significant since, as we explain in the main Article all instances of integral feedback control reported in the literature to date use what we refer to here as a single opposer node. But here we see that single opposer nodes are simply special cases of a more general topological phenomenon that we refer to as an *opposing set*. Hereafter, we will consider a single opposer node to be a *trivial opposing set*.

SN3.2.3 Internally Valid M-sets

What properties must a collection of m non-zero elements of $\mathscr{R}$ possess in order to be able to form an internally valid M-set?

First, it is clear that an M-set must contain at least one positive term and one negative term in order for a solution to its LAQ to exist. Second, considering that every element of $\mathscr{R}$ has a route component and a cycle component (the latter being unity in the event that the associated route component contains all n nodes, and the former being unity if a single node acts as both input and output), there are two distinct logical possibilities for any multi-term subset of $\mathscr{R}$:

1. A single route (or no route) is represented in the subset. In this case, each term contains the same route (or no route at all) with a different cycle-product. Note that a particular cycle may be common to two or more cycle products, but each cycle-product taken as a whole must be unique to each term.

2. Multiple routes are represented.

From a consideration of these two possibilities arises the following theorem:

Theorem 4. *An internally valid M-set must contain more than one route.*

Proof. Suppose we have an internally valid M-set with either no route, or only one route. The associated LAQ can now be considered. From this LAQ, all common factors (being non-zero by hypothesis for an M-set) may be cancelled; in the case of a single route, this necessarily appears in every term, and the route therefore cancels from every term. Hence, each term of the LAQ, thus reduced, contains a unique combination of *cycle products*.

Now, consider every instance of a 1-cycle (ie. a factor of the form $\frac{\partial f_i}{\partial P_i}$ - a kinetic multiplier) in the reduced LAQ. From the k different 1-cycles appearing in the reduced LAQ ($0 \le k \le n$), we form the product $\Gamma = \prod_A \frac{\partial f_i}{\partial P_i}$, where $A \subset \{1, \ldots, n\}$ indicates the subset of nodes whose kinetic multipliers appear in the reduced LAQ; when there are no 1-cycles in the reduced LAQ ($k = 0$), $\Gamma = 1$.

We now divide every term of the reduced LAQ by Γ, to obtain a ‘modified’ LAQ which comprises *only circuit links*, each scaled by the kinetic multiplier of the terminal node for the link. Now observe that (1) the reaction kinetics governing any link are independent of the reaction kinetics governing any other link, and (2) that each term in the reduced and modified LAQ corresponds to a *unique* circuit combination.

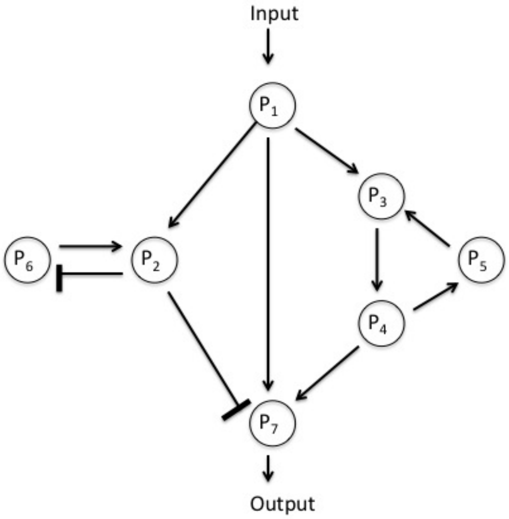

Supplementary Figure 1: Schematic diagram for the simple 7-node network used for Example 1.

Thus, the sum of the terms of the reduced/modified LAQ (and consequently of the original LAQ) cannot be identically zero for all $\mathscr{I}$ and all π_n, and the M-set is not internally valid - a contradiction.

Thus, an internally valid M-set must contain more than one route. □

Corollary 2. *For networks in which the input node and the output node are one and the same node, an M-set cannot feature in the partition of $\mathscr{R}$ into MA-subsets.*

The following simple example provides a concrete illustration of the essential principles underlying Theorem 4.

Example 1. Supplementary Figure 1 depicts a schematic diagram for a simple 7-node network.

The RPA equation for this network is given by

$$\begin{aligned}&\frac{\partial f_7}{\partial P_1}\frac{\partial f_2}{\partial P_6}\frac{\partial f_6}{\partial P_2}\frac{\partial f_3}{\partial P_3}\frac{\partial f_4}{\partial P_4}\frac{\partial f_5}{\partial P_5}+\frac{\partial f_7}{\partial P_1}\frac{\partial f_2}{\partial P_6}\frac{\partial f_6}{\partial P_2}\frac{\partial f_3}{\partial P_5}\frac{\partial f_4}{\partial P_3}\frac{\partial f_5}{\partial P_4}-\frac{\partial f_7}{\partial P_1}\frac{\partial f_2}{\partial P_2}\frac{\partial f_6}{\partial P_6}\frac{\partial f_3}{\partial P_5}\frac{\partial f_4}{\partial P_3}\frac{\partial f_5}{\partial P_4}\\&-\frac{\partial f_7}{\partial P_1}\frac{\partial f_2}{\partial P_2}\frac{\partial f_6}{\partial P_6}\frac{\partial f_3}{\partial P_3}\frac{\partial f_4}{\partial P_4}\frac{\partial f_5}{\partial P_5}+\frac{\partial f_2}{\partial P_1}\frac{\partial f_7}{\partial P_2}\frac{\partial f_6}{\partial P_6}\frac{\partial f_3}{\partial P_3}\frac{\partial f_4}{\partial P_4}\frac{\partial f_5}{\partial P_5}+\frac{\partial f_2}{\partial P_1}\frac{\partial f_7}{\partial P_2}\frac{\partial f_6}{\partial P_6}\frac{\partial f_3}{\partial P_5}\frac{\partial f_4}{\partial P_3}\frac{\partial f_5}{\partial P_4}\\&-\frac{\partial f_3}{\partial P_1}\frac{\partial f_4}{\partial P_3}\frac{\partial f_7}{\partial P_4}\frac{\partial f_2}{\partial P_2}\frac{\partial f_5}{\partial P_5}\frac{\partial f_6}{\partial P_6}+\frac{\partial f_3}{\partial P_1}\frac{\partial f_4}{\partial P_3}\frac{\partial f_7}{\partial P_4}\frac{\partial f_5}{\partial P_5}\frac{\partial f_2}{\partial P_6}\frac{\partial f_6}{\partial P_2}=0.\end{aligned}$$

We consider taking the first three terms of this equation as a possible M-set; we note that all three of these terms have the same route. The LAQ for this potential M-set is thus

$$\frac{\partial f_7}{\partial P_1}\frac{\partial f_2}{\partial P_6}\frac{\partial f_6}{\partial P_2}\frac{\partial f_3}{\partial P_3}\frac{\partial f_4}{\partial P_4}\frac{\partial f_5}{\partial P_5}+\frac{\partial f_7}{\partial P_1}\frac{\partial f_2}{\partial P_6}\frac{\partial f_6}{\partial P_2}\frac{\partial f_3}{\partial P_5}\frac{\partial f_4}{\partial P_3}\frac{\partial f_5}{\partial P_4}-\frac{\partial f_7}{\partial P_1}\frac{\partial f_2}{\partial P_2}\frac{\partial f_6}{\partial P_6}\frac{\partial f_3}{\partial P_5}\frac{\partial f_4}{\partial P_3}\frac{\partial f_5}{\partial P_4}=0 \tag{7}$$

Let us assume that all kinetic multipliers are negative for this network, and that the links are activating or inhibitory in nature as indicated in the diagram. The signs preceding each of the three terms in Equation 7 are native signs. The influence sign of the first term will be positive since there are a total of four negative

cycles - three (negative) kinetic multipliers along with the negative circuit between nodes 2 and 6. The influence sign of the second term will be negative, since it contains one negative cycle - the circuit between nodes 2 and 6. The influence sign of the third term will be positive since it contains two negative kinetic multipliers and the positive cycle among nodes 3, 4 and 5. With these *overall* signs taken into account, the terms may be distributed to the two sides of the equation as follows:

$$\left|\frac{\partial f_7}{\partial P_1}\frac{\partial f_2}{\partial P_6}\frac{\partial f_6}{\partial P_2}\frac{\partial f_3}{\partial P_3}\frac{\partial f_4}{\partial P_4}\frac{\partial f_5}{\partial P_5}\right| = \left|\frac{\partial f_7}{\partial P_1}\frac{\partial f_2}{\partial P_6}\frac{\partial f_6}{\partial P_2}\frac{\partial f_3}{\partial P_5}\frac{\partial f_4}{\partial P_3}\frac{\partial f_5}{\partial P_4}\right| + \left|\frac{\partial f_7}{\partial P_1}\frac{\partial f_2}{\partial P_2}\frac{\partial f_6}{\partial P_6}\frac{\partial f_3}{\partial P_5}\frac{\partial f_4}{\partial P_3}\frac{\partial f_5}{\partial P_4}\right|. \tag{8}$$

Now, each term may be divided through by the common (route) factor, $\frac{\partial f_7}{\partial P_1}$, and by the product $\frac{\partial f_2}{\partial P_2}\frac{\partial f_3}{\partial P_3}\frac{\partial f_4}{\partial P_4}\frac{\partial f_5}{\partial P_5}\frac{\partial f_6}{\partial P_6}$, thus yielding the reduced LAQ,

$$\left|\frac{\frac{\partial f_2}{\partial P_6}\frac{\partial f_6}{\partial P_2}}{\frac{\partial f_2}{\partial P_2}\frac{\partial f_6}{\partial P_6}}\right| = \left|\frac{\frac{\partial f_3}{\partial P_5}\frac{\partial f_4}{\partial P_3}\frac{\partial f_5}{\partial P_4}}{\frac{\partial f_3}{\partial P_3}\frac{\partial f_4}{\partial P_4}\frac{\partial f_5}{\partial P_5}}\right| + \left|\frac{\frac{\partial f_2}{\partial P_6}\frac{\partial f_6}{\partial P_2}\frac{\partial f_3}{\partial P_5}\frac{\partial f_4}{\partial P_3}\frac{\partial f_5}{\partial P_4}}{\frac{\partial f_2}{\partial P_2}\frac{\partial f_6}{\partial P_6}\frac{\partial f_3}{\partial P_3}\frac{\partial f_4}{\partial P_4}\frac{\partial f_5}{\partial P_5}}\right|. \tag{9}$$

We thus see that each term constitutes a unique set of circuits, with each component nodal reaction (link) scaled by its own kinetic multiplier. Since each such nodal reaction rate is a function that is independent of all other nodal reaction rates, involving independent parameters, the two sides of the equation cannot be identified for all $\mathscr{I}$ and all π_n, except in the special case of tuned parameters. RPA is, by definition, perfect adaptation to a stimulus, $\mathscr{I}$, that does not require special tuning of parameters.

□

Thus, the problem at hand reduces to determining the characteristics of a multi-route multi-term subset of $\mathscr{R}$ with a solvable LAQ of the form

$$|\Sigma S^{+}| = |\Sigma S^{-}|,$$

where $|\Sigma S^{+}|$ represents the sum of all positive terms in the set, and $|\Sigma S^{-}|$ represents the absolute value of the sum of all negative terms in the set.

Now, in general, the terms of the LAQ will contain common factors - route links, circuit links and kinetic multipliers - that may be cancelled from the LAQ (provided they are non-zero[1] in the case of kinetic multipliers) to produced a 'reduced' LAQ (hereafter, an rLAQ), of the form

$$|\Sigma \hat{S}^{+}| = |\Sigma \hat{S}^{-}|. \tag{10}$$

To determine which factors are common to a particular collection of terms, and their topological role in the associated network, consider first the following argument: Each of the terms in the subset contains a route component, such that ρ routes ($2 \le \rho \le m$) are represented in the subset. All ρ routes necessarily share *at least* the input node P_i and the output node P_j. Thus, there will exist two nodes of special topological significance to any candidate M-set:

1. A 'diverter' node (hereafter, the 'D-node' for the subset in question), being the most downstream node common to all routes in the subset, having the property that all nodes upstream of it are also common to all routes in the subset; and
2. A 'connector' node (hereafter, the 'C-node' for the subset in question), being the most upstream node common to all routes, having the property that all nodes downstream of it are also common to all routes.

Thus, to any collection of terms (representing a proposed M-set) may be associated a unique pair of nodes, a D-node and a C-node, ie. a D-C node pair.

[1] Note that zero-valued kinetic multipliers form S-sets, thereby removing the associated terms from the proposed M-set; the terms of an M-set are non-zero by definition.

We remark in passing that in general, if the proposed M-set is sufficiently large, there could be additional node(s), at intermediate location(s) between the D-node and the C-node, that are also common to all routes in the M-set; in cases such as these, we will see in Supplementary Note 5 that such a set is not technically *minimally adaptive* since it can be subdivided into smaller subsets that can satisfy a LAQ independently of the remaining terms. For this reason, we initially assume *a priori* that the otherwise arbitrary M-set under consideration contains no nodes between the D-node and the C-node that are common to all routes in the set.

In addition, for clarity of exposition, we consider in the first instance that any route present in the proposed M-set is *fully represented* in that M-set; that is, all instances of the given route (with the various possible cycle combinations that exist for that route) will also be absorbed into the M-set. (In Supplementary Note 5, the possibilities and consequences of having instances of a particular route distributed to multiple MA-subsets (ie. to both the M-set and its complement in $\mathscr{R}$) will be clarified through the notion of the union of M-sets to form "RPA basis elements".)

Now, we observe that the D-C node pair delineates which factors are common to all m terms in the proposed M-set, and which are contained in only a subset of the m terms. In particular:

1. All links above D and below C are common to all m terms;
2. Network circuits that incorporate *any* node(s) above and including D, or below and including C, will not appear in any of the m terms, since they are not disjoint from any route included in the subset;
3. For any network circuit that is disjoint from *all* of the routes in the subset, the nodes comprising the circuit will appear within the cycle component of all m terms. If the network circuit contains an opposer node, then the circuit itself will be common to all m terms; otherwise, if the circuit does not contain an opposer, each term is duplicated with the product of the kinetic multipliers of the circuit nodes appearing instead of the circuit itself containing those same nodes. Either way, the factors containing the circuit nodes will cancel from the LAQ.
4. Kinetic multipliers for non-circuit nodes that are disjoint from *all* of the routes in the subset are common to all m terms.

It follows from these four properties taken together that the terms of the rLAQ represent chains of network links commencing at the D-node and terminating at the C-node, together with cycle-products comprising combinations of: (a) kinetic multipliers for all nodes participating in route-portions connecting D and C (not inclusive, since D and C are common to the routes in question), and (b) circuits that are entirely embedded in these chains of links (that is, not containing D or anything upstream of it, or C or anything downstream of it).

We thus conclude that the only nodes whose 'regulations' appear in the rLAQ are those located interior to the D-C node pair, (hereafter referred to as the 'interior nodes' for the subset in question), along with the C-node itself. Note that 'interior nodes' are those included in the portions of the routes contained between D and C (exclusive of D but inclusive of C), as well as those that are members of network circuits that are fully embedded in these route-portions. These considerations imply a well-defined network module associated with M-sets, which we refer to hereafter as a *Balancer Module* and which we depict in Supplementary Figure 2.

SN3.2.4 Creation of M-sets in the Network

We now consider under what circumstances, biochemically speaking (in terms of constraints on reaction kinetics), Equation (10) has a solution, such that the associated terms from the RPA equation "balance" to form an internally-valid M-set.

We note that the terms are distributed to the two sides of Equation (10) according to the sign of each term. We do note in Supplementary Note 8 that the influence signs of the cycles in the terms of the RPA equation play a role in the stability of the (perfectly-adaptive) steady-state. In particular, while we show that it theoretically possible under very special (parametric) circumstances for a network to contain a positive cycle and achieve a (locally) asymptotically stable steady-state, we see that the presence of even

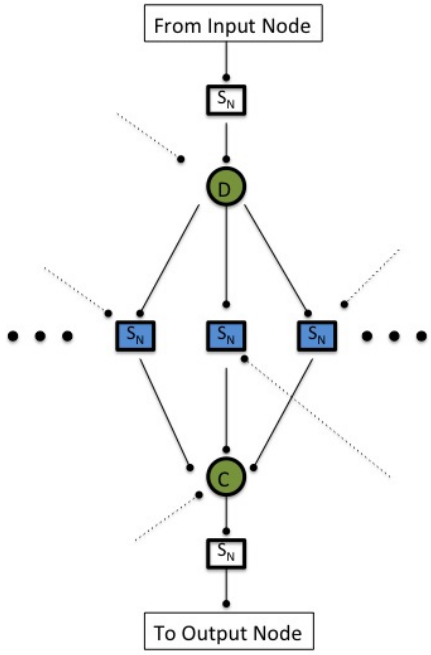

Supplementary Figure 2: General form of a Balancer Module, characterised by a D-C node pair (indicated in green), and an arbitrary number of interior nodes, at least one, indicated in blue. The elements S_N indicate that any arbitrary sub-network or module may be fully embedded in the indicated positions. Dashed regulations from the outside indicate that the indicated network elements may, in principle, be regulated by nodes outside the module. We will see in Section that these outside regulations are *only* valid if they are "adapted nodes", due to the activities of other modules in the wider network. The three dots on either side of the interior S_N elements indicate that a module may contain any arbitrary number of route-segments between the D-node and the C-node; three overarching route segments are indicated for illustrative purposes.

one positive cycle is highly conducive to steady-state instability, and is thus not a generic property of solutions to the RPA problem.

We note, moreover, that if all network cycles are negative (which will generically be true of stable solutions to the RPA problem), Corollary 1 in Supplementary Note 2 indicates that all copies of a given route in a proposed M-set will appear on the same side of Equation (10). For clarity of exposition, then, we will hereafter consider expressions for rLAQs in which instances of a given route will not be distributed to both sides of the equation; we thus require that at least one route in the M-set be negative (containing an odd number of inhibitory links), and at least one route be positive (with an even number of inhibitory links), in order to have the potential to form an internally-valid M-set. (We emphasise, however, that in special cases involving positive cycle(s), where instances of a route do appear on both sides of the equation, this does not - in and of itself - prevent the formation of a valid M-set. While such a situation is highly conducive to an unstable (perfectly-adaptive) steady-state, this scenario may theoretically produce a valid solution to the RPA problem; in such a case, the positivity of a cycle may allow routes in the proposed M-set to be either all negative or all positive).

With these considerations in mind, we now examine in more detail the contents of the rLAQ for a proposed M-set. We begin by proposing the following definitions:

Definition 14. *Interior nodes: In the context of M-sets, and their associated Balancer Modules, an interior node is any node that is either included strictly within the route segment between the D-node and the C-node associated to the M-set, or a member of a network circuit that is fully embedded in such a route segment. All interior nodes are thus necessarily connected and transmissive with respect to the D-node/C-node pair of the Balancer module.*

Definition 15. *Terminal link: In the context of M-sets, and their associated Balancer Modules, a terminal link is any regulation expressed by the factor* $\frac{\partial f_C}{\partial P_i}$*, where* P_C *is the C-node for the M-set, and* P_i *is an interior node immediately upstream of the C-node.*

We must now consider how (or if) a proposed M-set can create an internally-valid MA-subset - that is, how (or if) Equation (10) has a solution for the selected set of terms. Now, we can see that each term of the rLAQ associated to an M-set is a product of a route segment (from D-node to C-node) and a cycle product. Each such route segment is comprised of a succession of links involving interior nodes and culminates in a *terminal link* (consisting of the regulation of the C-node by the immediately upstream interior node for that route segment). Each cycle product appended to the route segment is a *unique* combination of cycles involving *all interior* nodes - kinetic multipliers and circuits involving (only) interior nodes. Kinetic multipliers for nodes other than interior nodes will not appear in the rLAQ: kinetic multipliers for nodes that are disjoint from all routes contained that in the M-set will have cancelled in obtaining the rLAQ from the LAQ; kinetic multipliers for nodes that are upstream of the D-node (inclusive of the D-node itself) or downstream of the C-node (inclusive of the C-node itself) will not appear in the terms of the M-set, since they are 'route nodes' whose kinetic multipliers can therefore not appear in the cycle components of the terms. Similarly, network circuits that are disjoint from *all* routes of the M-set cannot appear in the rLAQ since they will have 'cancelled'; in addition, any circuit that is contiguous with all routes (ie. circuits containing any node upstream of the D-node, inclusive, or downstream of the C-node, inclusive) will not appear in the cycle components of the terms of the M-set.

Now, a taking account of the uniqueness of these route-segment/cycle products, along with the fact that the regulation of any given node f_a is independent of the regulation of any other node f_b, $a \neq b$, yields the following theorem:

Theorem 5. *For an internally valid M-set, every interior node must have a steady-state value that is linearly related to the steady-state value of every other interior node, as well as that of the D-node. That is, the projection of the network's steady-state trajectory onto the* b*-dimensional subspace of* $\mathbb{R}^n$ *associated with the* b *interior nodes is a straight line parametrized by the steady-state value of the D-node.*

Proof. We have established that each term of the rLAQ is a unique route-segment/cycle-product combination comprising *only interior nodes* for the M-set under consideration.

We consider the product, $\Gamma = \prod_{A} \frac{\partial f_i}{\partial P_i}$, where $A \subset \{1,\ldots,n\}$ indicates the set of *all interior* nodes for the M-set. (In other words, Γ represents the product of all kinetic multipliers for the set of all interior nodes in the M-set).

We now divide every term of the rLAQ by Γ, to obtain a 'modified' rLAQ; each term of this modified rLAQ thus comprises the product of a terminal link with a unique set of (*interior*) nodal regulations (links); moreover, each such interior nodal regulation is scaled by its *own* kinetic multiplier. That is, each internal node, P_i will now appear in the modified rLAQ in the form $\frac{\frac{\partial f_i}{\partial P_k}}{\frac{\partial f_i}{\partial P_i}}$, where P_k is the node regulating P_i in the circuit or route-segment in question.

Moreover, the nodal reaction rate for every node is a function that is independent of any other nodal reaction rate, involving independent parameters (and generally, different reactants); thus, the reaction kinetics at each node are necessarily independent of the kinetics at every other node. In other words, the functions, f_i, corresponding to each internal node are independent functions. The possibility of satisfying the rLAQ (10), and hence the LAQ, *for all* $\mathscr{I}$ *and all* π_n thus requires

$$\frac{\frac{\partial f_i}{\partial P_k}}{\frac{\partial f_i}{\partial P_i}} = K_{ij}, \tag{11}$$

for each interior node, P_i, and each of its interior (or D-node) regulators, P_k, where $K_{ij} \in \mathbb{R}$ is a 'constant' (a parameter, which may be positive or negative according to the nature of the regulations involved in the quotient on the left-hand side of (11)) that is independent of the steady-state values of all interior nodes. K_{ij} could vary with the steady-state values of *other* network nodes, outside the Balancer Module, however, should interior node(s) have regulators from amongst those other nodes. (This, of course, would require those outside regulators to have a "fixed" steady-state value (independent of $\mathscr{I}$); in other words, they must exhibit the RPA property due to the influence of modules generated by the complement in $\mathscr{R}$ of the M-set).

Now, Equation (11) implies that

$$\frac{\partial P_i^*}{\partial P_k^*} = -K_{ij}, \tag{12}$$

for all $\mathscr{I}$ and all π_n, where the superposed asterixes indicate steady-state values (recalling that the partial derivatives appearing in the RPA equation, and all relations and equations derived from it, are to be evaluated at the system steady states). Since Equation (12) must hold for the regulation of each interior node of the M-set, P_i, by each interior (or D-node) regulator in the M-set, and since interior nodes are both connected and transmissive with respect to their associated D-node/C-node pair, it follows that the steady-states of all interior nodes (as well as that of the D-node) are all linearly dependent (with 'constants', K_{ij}, which could in principle be affected by the steady-states of nodes *outside* the balancer module in question).

Thus, every interior node must have a steady-state value that is linearly related to the steady-state value of every other interior node, as well as that of the D-node, for that M-set. □

For this reason, we refer to the internal nodes of an M-set hereafter as *balancer nodes* on account of the constraints on their reaction kinetics in order to create the linear relationships among nodal steady-states prescribed by Theorem 5. These requisite reaction kinetics, elaborated in Supplementary Note 4, will be referred to hereafter as *balancer kinetics*. Moreover, Theorem 5 implies that Equation (10) must assume the following general form:

$$\sum_i \hat{K}_i \frac{\partial f_C}{\partial P_i} = \sum_j \hat{K}_j \frac{\partial f_C}{\partial P_j}, \tag{13}$$

where the indices i and j represent pre-terminal nodes (ie. immediately upstream of the C-node) for the positive and negative route segments, respectively. (We note again that for clarity of illustration, we have presented the general form that corresponds to the scenario where all cycles are negative; existence of a positive cycle may distribute terms associated with a single terminal link to both sides of the equation.)

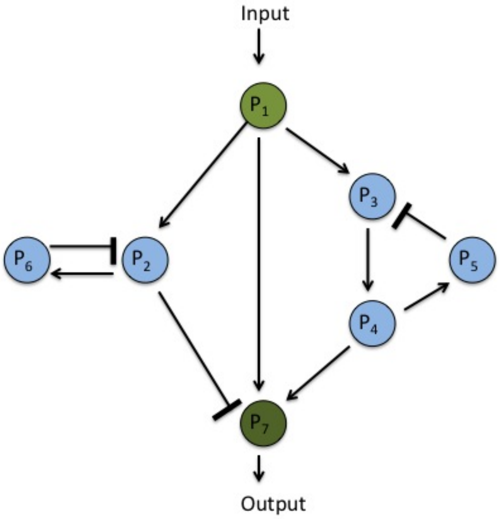

Supplementary Figure 3: Network schematic for the simple 7-node network analysed in Example 2. The D-C node pair is indicated in green; the interior nodes are indicated in blue.

Now, satisfying Equation (13) for all $\mathscr{I}$ and all π_n, requires

$$P_C^* = \hat{K}, \tag{14}$$

where $\hat{K}$ is a constant of the M-set, being independent of the steady-states of its internal nodes. This, in turn, places constraints on the reaction kinetics at the C-node, P_C. We will refer to the requisite reaction kinetics at the C-node hereafter as *connector kinetics*. The mechanisms underlying connector kinetics will be elaborated in Section .

By way of a simple concrete example to illustrate the essential principles underlying Theorem 5, we consider once again the simple 7-node network considered in Example 1. Unlike that example, however, we now consider the link from P_5 to P_3 to be an inhibitory one, enabling the cycleP_3-P_4-P_5-P_3 to be a stability-promoting negative one . (Recall that in the previous example (Example 1) we required a positive cycle P_3-P_4-P_5-P_3 in order to be able to distribute instances of the single route P_1-P_7 to both sides of the associated LAQ for that case).

Example 2. Supplementary Figure 3 above presents the schematic diagram for this 7-node network.

We saw in Example 1 that the RPA equation for this network is given by:

$$\frac{\partial f_7}{\partial P_1}\frac{\partial f_2}{\partial P_6}\frac{\partial f_6}{\partial P_2}\frac{\partial f_3}{\partial P_3}\frac{\partial f_4}{\partial P_4}\frac{\partial f_5}{\partial P_5}+\frac{\partial f_7}{\partial P_1}\frac{\partial f_2}{\partial P_6}\frac{\partial f_6}{\partial P_2}\frac{\partial f_3}{\partial P_5}\frac{\partial f_4}{\partial P_3}\frac{\partial f_5}{\partial P_4}-\frac{\partial f_7}{\partial P_1}\frac{\partial f_2}{\partial P_2}\frac{\partial f_6}{\partial P_6}\frac{\partial f_3}{\partial P_5}\frac{\partial f_4}{\partial P_3}\frac{\partial f_5}{\partial P_4}$$
$$-\frac{\partial f_7}{\partial P_1}\frac{\partial f_2}{\partial P_2}\frac{\partial f_6}{\partial P_6}\frac{\partial f_3}{\partial P_3}\frac{\partial f_4}{\partial P_4}\frac{\partial f_5}{\partial P_5}+\frac{\partial f_2}{\partial P_1}\frac{\partial f_7}{\partial P_2}\frac{\partial f_6}{\partial P_6}\frac{\partial f_3}{\partial P_3}\frac{\partial f_4}{\partial P_4}\frac{\partial f_5}{\partial P_5}+\frac{\partial f_2}{\partial P_1}\frac{\partial f_7}{\partial P_2}\frac{\partial f_6}{\partial P_6}\frac{\partial f_3}{\partial P_5}\frac{\partial f_4}{\partial P_3}\frac{\partial f_5}{\partial P_4}$$
$$-\frac{\partial f_3}{\partial P_1}\frac{\partial f_4}{\partial P_3}\frac{\partial f_7}{\partial P_4}\frac{\partial f_2}{\partial P_2}\frac{\partial f_5}{\partial P_5}\frac{\partial f_6}{\partial P_6}+\frac{\partial f_3}{\partial P_1}\frac{\partial f_4}{\partial P_3}\frac{\partial f_7}{\partial P_4}\frac{\partial f_5}{\partial P_5}\frac{\partial f_2}{\partial P_6}\frac{\partial f_6}{\partial P_2}=0.$$

We consider all eight terms in the RPA equation to be non-zero (no kinetic multipliers identically zero at steady-state), and for simplicity, consider the possibility of forming a single M-set from all eight terms.

The D-node for the M-set is thus the input node, P_1, and the C-node is the output node, P_7. We further assume that all kinetic multipliers are negative, and we note from the diagram that the two circuits in the network are negative; thus, for this example, all cycles are negative.

With the net sign for each term thus determined, the LAQ for this proposed M-set is:

$$\begin{aligned}&\left|\frac{\partial f_7}{\partial P_1}\frac{\partial f_2}{\partial P_6}\frac{\partial f_6}{\partial P_2}\frac{\partial f_3}{\partial P_3}\frac{\partial f_4}{\partial P_4}\frac{\partial f_5}{\partial P_5}\right| + \left|\frac{\partial f_7}{\partial P_1}\frac{\partial f_2}{\partial P_6}\frac{\partial f_6}{\partial P_2}\frac{\partial f_3}{\partial P_5}\frac{\partial f_4}{\partial P_3}\frac{\partial f_5}{\partial P_4}\right| + \left|\frac{\partial f_7}{\partial P_1}\frac{\partial f_2}{\partial P_2}\frac{\partial f_6}{\partial P_6}\frac{\partial f_3}{\partial P_5}\frac{\partial f_4}{\partial P_3}\frac{\partial f_5}{\partial P_4}\right|\\
&+\left|\frac{\partial f_7}{\partial P_1}\frac{\partial f_2}{\partial P_2}\frac{\partial f_6}{\partial P_6}\frac{\partial f_3}{\partial P_3}\frac{\partial f_4}{\partial P_4}\frac{\partial f_5}{\partial P_5}\right| + \left|\frac{\partial f_3}{\partial P_1}\frac{\partial f_4}{\partial P_3}\frac{\partial f_7}{\partial P_4}\frac{\partial f_2}{\partial P_2}\frac{\partial f_5}{\partial P_5}\frac{\partial f_6}{\partial P_6}\right| + \left|\frac{\partial f_3}{\partial P_1}\frac{\partial f_4}{\partial P_3}\frac{\partial f_7}{\partial P_4}\frac{\partial f_5}{\partial P_5}\frac{\partial f_2}{\partial P_6}\frac{\partial f_6}{\partial P_2}\right|\\
&= \left|\frac{\partial f_2}{\partial P_1}\frac{\partial f_7}{\partial P_2}\frac{\partial f_6}{\partial P_6}\frac{\partial f_3}{\partial P_3}\frac{\partial f_4}{\partial P_4}\frac{\partial f_5}{\partial P_5}\right| + \left|\frac{\partial f_2}{\partial P_1}\frac{\partial f_7}{\partial P_2}\frac{\partial f_6}{\partial P_6}\frac{\partial f_3}{\partial P_5}\frac{\partial f_4}{\partial P_3}\frac{\partial f_5}{\partial P_4}\right|.\end{aligned}$$

Moreover, we can produce from this LAQ a 'modified' rLAQ of the following form:

$$\begin{aligned}&\left(\left|\frac{\frac{\partial f_2}{\partial P_6}\frac{\partial f_6}{\partial P_2}}{\frac{\partial f_2}{\partial P_2}\frac{\partial f_6}{\partial P_6}}\right| + \left|\frac{\frac{\partial f_2}{\partial P_6}\frac{\partial f_6}{\partial P_2}\frac{\partial f_3}{\partial P_5}\frac{\partial f_4}{\partial P_3}\frac{\partial f_5}{\partial P_4}}{\frac{\partial f_2}{\partial P_2}\frac{\partial f_6}{\partial P_6}\frac{\partial f_3}{\partial P_3}\frac{\partial f_4}{\partial P_4}\frac{\partial f_5}{\partial P_5}}\right| + \left|\frac{\frac{\partial f_3}{\partial P_5}\frac{\partial f_4}{\partial P_3}\frac{\partial f_5}{\partial P_4}}{\frac{\partial f_3}{\partial P_3}\frac{\partial f_4}{\partial P_4}\frac{\partial f_5}{\partial P_5}}\right| + 1\right)\frac{\partial f_7}{\partial P_1} + \left(\left|\frac{\frac{\partial f_3}{\partial P_1}\frac{\partial f_4}{\partial P_3}}{\frac{\partial f_3}{\partial P_3}\frac{\partial f_4}{\partial P_4}}\right| + \left|\frac{\frac{\partial f_3}{\partial P_1}\frac{\partial f_4}{\partial P_3}\frac{\partial f_2}{\partial P_6}\frac{\partial f_6}{\partial P_2}}{\frac{\partial f_3}{\partial P_3}\frac{\partial f_4}{\partial P_4}\frac{\partial f_2}{\partial P_2}\frac{\partial f_6}{\partial P_6}}\right|\right)\frac{\partial f_7}{\partial P_4}\\
&= \left(\left|\frac{\frac{\partial f_2}{\partial P_1}}{\frac{\partial f_2}{\partial P_2}}\right| + \left|\frac{\frac{\partial f_2}{\partial P_1}\frac{\partial f_3}{\partial P_5}\frac{\partial f_4}{\partial P_3}\frac{\partial f_5}{\partial P_4}}{\frac{\partial f_2}{\partial P_2}\frac{\partial f_3}{\partial P_3}\frac{\partial f_4}{\partial P_4}\frac{\partial f_5}{\partial P_5}}\right|\right)\left|\frac{\partial f_7}{\partial P_2}\right|.\end{aligned}$$

We thus see that each term comprises the product of a terminal link with a *unique* combination of regulations of a subset of the interior nodes, $\{P_2, P_3, P_4, P_5, P_6\}$, each regulation (link) being scaled by the kinetic multiplier of the regulated node. In addition, the functions f_2, f_3, f_4, f_5 and f_6 are independent functions, encoding the regulations of independent nodes, so we require each 'scaled regulation' in the above rLAQ to be of the form, $\frac{\frac{\partial f_i}{\partial P_j}}{\frac{\partial f_i}{\partial P_i}} = K_{ij} \in \mathbb{R}$ ($i \neq j$, $i \in \{2,3,4,5,6\}$, $j \in \{1,2,3,4,5,6\}$; this in turn requires reaction kinetics for f_i, $i \in 2,3,4,5,6$ such that $\frac{\partial P_i^*}{\partial P_j^*} = -K_{ij}$. Thus, the rLAQ will reduce to the general form

$$\kappa_a \frac{\partial f_7}{\partial P_1} + \kappa_b \frac{\partial f_7}{\partial P_4} = \kappa_c \left|\frac{\partial f_7}{\partial P_2}\right|, \tag{15}$$

where the various constants, K_{ij} have now been absorbed into the general constants $\kappa_a, \kappa_b, \kappa_c$. Satisfying Equation (15) for all $\mathscr{I}$ and all π_n, thus requires a suitable form for f_7, giving

$$P_7^* = \hat{\kappa}.$$

By way of illustration, we propose a simple example forms for the reaction kinetics of each node in this 7-node network, and simulate the time-dependent behaviour of several nodes as the input to the network gradually increases in a step-fashion. See Supplementary Figure 4. From the above argument, we see that there are no constraints on the functional form of f_1, the input node and D-node of the single M-set. For the interior nodes, and the C-node, we see that the following simple functional forms meet the criteria

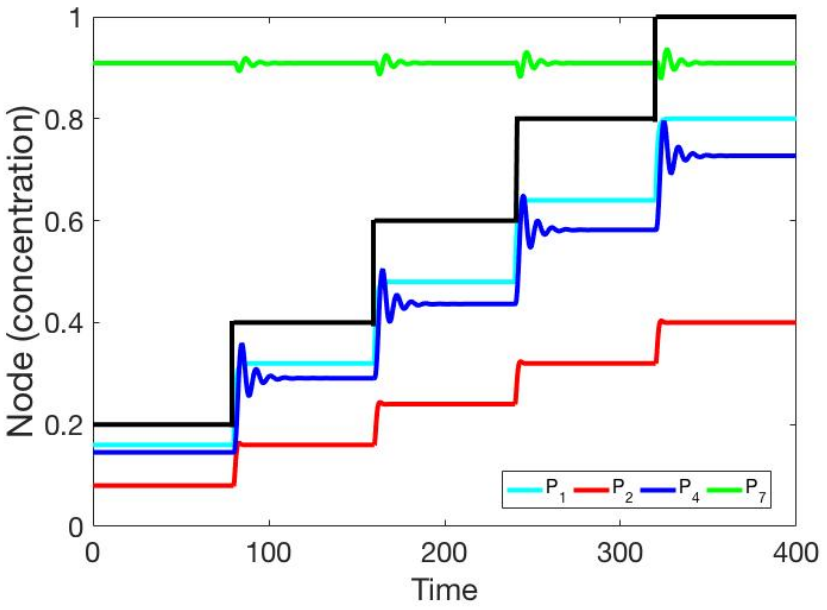

Supplementary Figure 4: Time-dependent simulations for the simple 7-node network depicted in Supplementary Figure 3. Equations are as given in Eqs (16) through (22). Input, $\mathscr{I}$ is indicated by the black step function: its value begins at 0.2, and increases in increments of 0.2 until a value of 1. The time-dependent response of the input node (D-node), P_1, as well as interior nodes P_2 and P_4 are depicted, and shown not to adapt to the varying input; similarly the other interior nodes, P_3, P_5 and P_6 (not shown), do not adapt to the input. The output node (C-node), P_7, depicted in green, does adapt to the varying input. Parameters are: $k_1 = 0.8$; $k_2 = k_3 = k_4 = k_5 = k_6 = k_7 = k_8 = k_9 = k_{11} = k_{12} = k_{13} = k_{14} = k_{15} = 1$; $k_{10} = 0.1$; $k_{16} = 0.5$; $k_{17} = 2$.

above for *balancer kinetics* and *connector kinetics*:

$$f_1 = \frac{dP_1}{dt} = k_1\mathscr{I} - k_2P_1, \tag{16}$$

$$f_2 = \frac{dP_2}{dt} = k_3P_1 - k_4P_6 - k_5P_2, \tag{17}$$

$$f_3 = \frac{dP_3}{dt} = k_6P_1 - k_7P_5 - k_8P_3, \tag{18}$$

$$f_4 = \frac{dP_4}{dt} = k_9P_3 - k_{10}P_4, \tag{19}$$

$$f_5 = \frac{dP_5}{dt} = k_{11}P_4 - k_{12}P_5, \tag{20}$$

$$f_6 = \frac{dP_6}{dt} = k_{14}P_2 - k_{15}P_6, \tag{21}$$

$$f_7 = \frac{dP_7}{dt} = k_{16}P_1P_4 - k_{17}P_2^2P_7. \tag{22}$$

Supplementary Note 4: The Chemical Basis of Robust Perfect Adaptation - Constraints on Reaction Kinetics

Here we examine the three types of 'special' nodes that underpin RPA: "opposer nodes", which arise in Opposer Modules, and "balancer nodes" and "connector nodes", which arise in Balancer Modules. As we shall see, the reaction kinetics required for each of these three node types are distinct (ie. mutually exclusive). This will form the governing principle for the creation of *relatively valid* MA-subsets, since a node that plays one role in a proposed MA-subset (say, an opposer node) cannot then play a different role (say, a balancer node) in the complement (in $\mathscr{R}$) of that subset.

SN4.1 S-sets: The Reaction Kinetics of Opposer Nodes

The reaction kinetics required for some node, P_o, to act as an opposer node are fully described by the constraint, $\frac{\partial f_o}{\partial P_o} = 0$. We now consider the general principles by which this constraint is implemented at a node.

Consider a candidate opposer node, P_o, whose activity is upregulated by some set of network nodes, $\{P_u\}$, and downregulated by some (other) set of network nodes, $\{P_d\}$. In principle, one of the sets could also contain P_o itself, if P_o were to play an active auto-regulatory role.

Now the existence of a non-trivial steady state for each node is predicated upon a positive (activating/synthesizing) and negative (inhibitory/deactivating/degrading) component to its overall reaction rate. Thus, in this instance we require the reaction rate, f_o, for P_o to assume the general form,

$$f_o = f_o^+(P_o, \{P_u\}) - f_o^-(P_o, \{P_d\}). \tag{23}$$

At the steady state,

$$f_o^+(P_o, \{P_u\}) = f_o^-(P_o, \{P_d\}), \tag{24}$$

and, additionally,

$$\frac{\partial f_o^+}{\partial P_o} = \frac{\partial f_o^-}{\partial P_o}, \tag{25}$$

in order to permit RPA, where both sides of (25) are evaluated at the steady-state defined by (24).

Analytically, combining (24) and (25) yields:

$$\frac{1}{f_o^+}\frac{\partial f_o^+}{\partial P_o} = \frac{1}{f_o^-}\frac{\partial f_o^-}{\partial P_o}, \tag{26}$$

where here and after, all quantities (ie. all reaction rates and all derivatives) are taken to be evaluated at the steady state, unless noted otherwise. Now, (26) implies that

$$\frac{\partial}{\partial P_o}\ln f_o^+(P_o, \{P_u\}) = \frac{\partial}{\partial P_o}\ln f_o^-(P_o, \{P_d\}).$$

Integrating now yields

$$\ln f_o^+ = \ln f_o^- + g(\{P_u\}, \{P_d\}),$$

or, equivalently,

$$\frac{f_o^+}{f_o^-} = e^{g(\{P_u\},\{P_d\})} = \hat{g}(\{P_u\}, \{P_d\}). \tag{27}$$

Thus, to satisfy the opposer kinetics at (all) steady-states, π_n, the ratio of the positive and negative contributions to the overall reaction rate must be independent of P_o. This in turn requires that the functional form of the reaction rate, f_o, be separable in P_o, such that

$$f_o = h_o(P_o)g_o^+(\{P_u\}) - h_o(P_o)g_o^-(\{P_d\}). \tag{28}$$

In other words, the P_o-dependencies on the forward (f_o^+) and reverse (f_o^-) sides of the f_o equation must be commensurable. This principle imposes strict constraints on the types of reaction kinetics at P_o that can enable the node to function as an opposer.

Examples of appropriate reaction kinetics for an opposer node, could be, for example:

$$f_o = k_1 P_u - k_2, \tag{29}$$

or

$$f_o = k_1 - k_2 P_d, \tag{30}$$

or

$$f_o = k_1 P_u P_o - k_2 P_o, \tag{31}$$

or

$$f_o = k_1 (P_{Otot} - P_o) - k_2 P_d (P_{Otot} - P_o). \tag{32}$$

Equations (29) and (30) represent zero-order (in the substrate, P_o) regulations; Equation (31) encodes positive autoregulation by P_o, while Equation (32) encodes autoregulation of the reverse reaction by the inactive form ($P_{Otot} - P_o$), with P_{Otot} representing the (fixed) sum of the active (P_o) and inactive forms - a mass conservation constraint.

But how might these reaction forms actually be implemented by well-established rate laws for chemical reactions? Using Michaelis-Menten reaction kinetics as an example, Equations (29) to (32) could be *approximated*, respectively, by:

$$f_o = \frac{k_1 P_u (P_{Otot} - P_o)}{K_{m1} + (P_{Otot} - P_o)} - \frac{k_2 P_o}{K_{m2} + P_o}, \tag{33}$$

subject to the parameter constraints $K_{m1} << (P_{Otot} - P_o)$ and $K_{m2} << P_o$;

$$f_o = \frac{k_1 (P_{Otot} - P_o)}{K_{m1} + (P_{Otot} - P_o)} - \frac{k_2 P_d P_o}{K_{m2} + P_o}, \tag{34}$$

subject, likewise, to the parameter constraints $K_{m1} << (P_{Otot} - P_o)$ and $K_{m2} << P_o$;

$$f_o = \frac{k_1 P_u P_o (P_{Otot} - P_o)}{K_{m1} + (P_{Otot} - P_o)} - \frac{\hat{k}_2 P_o}{K_{m2} + P_o}, \tag{35}$$

subject to the parameter constraints $K_{m1} << (P_{Otot} - P_o)$ and $K_{m2} >> P_o$, and where $\hat{k}_2 = k_2 K_{m2}$; and

$$f_o = \frac{\hat{k}_1 (P_{Otot} - P_o)}{K_{m1} + (P_{Otot} - P_o)} - \frac{k_2 P_d P_o (P_{Otot} - P_o)}{K_{m2} + P_o}, \tag{36}$$

subject to the parameter constraints $K_{m1} >> (P_{Otot} - P_o)$ and $K_{m2} << P_o$, and where $\hat{k}_1 = k_1 K_{m1}$. In all these scenarios, a parameter constraint of the form $K_{mi} << P_i$, where P_i is the substrate in the associated enzyme-catalyzed reaction, corresponds to the enzyme being *saturated*, or close to saturation. A parameter constraint of the form $K_{mi} >> P_i$, on the other hand, corresponds to the enzyme being *far from saturation*.

Now, satisfying Equation (28) at the steady-state requires

$$g_o^+(\{P_u^*\}) = g_o^-(\{P_d^*\}), \tag{37}$$

which in turn requires that the union of the sets $\{P_u\}$ and $\{P_u\}$ contain *only one independent* node, $P_R \in \{P_u\} \cup \{P_d\}$; all other members of the sets must be strictly dependent on the one independent member, P_R.

From this, it follows that the independent regulator, P_R, *adapts* to the network stimulus $\mathscr{I}$ (that is, it *exhibits RPA*), with its (fixed) steady-state value satisfying

$$g_o^+(P_R^*) = g_o^-(P_R^*). \tag{38}$$

From this observation, it further follows that the opposer node itself, P_o, *cannot* adapt. Indeed, it is readily seen that the opposer node computes a time-integral of the "error" in its single independent regulator (relative to its prescribed "adaptive" steady-state value). Considering example (29) above, for instance, the steady state-value of the single upregulator, P_u, is given by

$$P_u^* = \frac{k_2}{k_1},$$

which means that

$$f_o = \frac{dP_o}{dt} = k_1(P_u - P_u^*),$$

so that

$$P_o = k_1 \int_{t_o}^{t_o+t} (P_u(\tau) - P_u^*) d\tau.$$

Similarly, using example (31) as an additional illustration, we obtain the form

$$\ln P_o = k_1 \int_{t_o}^{t_o+t} (P_u(\tau) - P_u^*) d\tau,$$

or

$$P_o = e^{\int_{t_o}^{t_o+t} k_1 (P_u(\tau) - P_u^*) d\tau}.$$

Importantly, the "fixed" value of P_R is determined by the f_o-reaction; it is, however, also specified by its "own" reaction, f_R (since P_R *cannot* adopt opposer kinetics, being an *adaptive* node). Therefore, the reaction f_R must be (at least indirectly) influenced by P_o in order to for P_R to actually achieve the value specified by the f_o reaction (thereby allowing the opposer kinetics to be implemented). This requires both P_R and its associated opposer node P_o to participate in a common circuit.

In Supplementary Note 3, we explored the topological requirements for opposing sets, and found that their opposer nodes are distributed to a collection of interlinked circuits that are disjoint from the route they collectively oppose. Now, we combine this with the requirement for *each* opposer node to have a single independent regulator, and we conclude that at least one of the opposers in the set must *also* participate in a circuit that is contiguous with the route they collectively oppose. In other words, an opposing set consists of a collection of disjoint circuits that are embedded into the feedback component of a circuit that is contiguous with the opposed route. In the special case that the opposing set consists of a single opposer node, this corresponds to a topology where the opposer participates in the feedback component of a circuit that is contiguous with the opposed route (with no participation in any disjoint circuits). These well-defined topological features imply a well-defined Module associated with the opposition mechanism. We depict the general structure of the Opposer Module, along with two particular concrete examples of opposing sets, in Supplementary Figures 5 and 6; one for the special case of a single opposer node (Supplementary Figure 5), and the other for another special case of a two-node opposing set (Supplementary Figure 6), being the smallest non-trivial case of an opposing set. A more general representation of the topology of an opposing set is given in the main paper (Figure 4).

SN4.2 M-sets: The Reaction Kinetics of Balancer Nodes

In Supplementary Note 3 we established that for any proposed M-set, we require steady-states of all balancer nodes of the associated balancer module to be linearly related to one another (Theorem 5). We now examine the reaction kinetics of a generic balancer node, P_B, to determine how this 'linearity constraint' may generally be satisfied.

As in the examination of opposer nodes in the preceding section, here again two sets of regulatory nodes are considered, at least one of which must be non-empty: $\{P_U\}$ is the set of nodes which upregulates P_B, and $\{P_D\}$ is the set which downregulates P_B. Thus, in general, the reaction rate, f_B, will assume the form

$$f_B = f_B^+(P_B, \{P_U\}) - f_B^-(P_B, \{P_D\}),$$

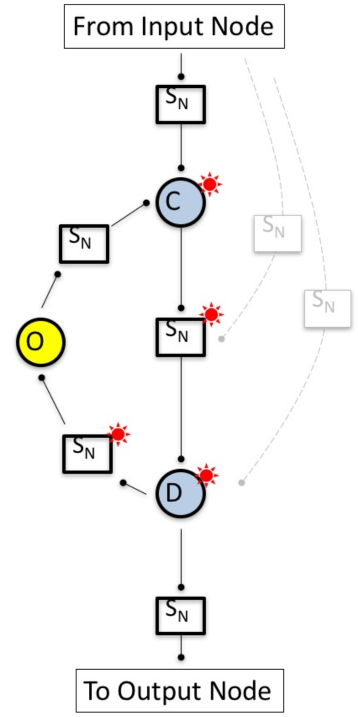

Supplementary Figure 5: General form of the Opposer Module generated by a single opposer node: As shown, the nodes "C" and "D" (both indicated in blue) delineate the feedback architecture of the module. The single opposer node is indicated by "O" in yellow. The red superposed asterisk indicates that the associated node(s) exhibit RPA due to the "opposing" influence of the single opposer node. The network element S_N indicates that any arbitrary subnetwork/motif/module may be fully embedded in these positions, without altering the RPA-generating function of the single opposer node; in other words the node upstream of S_N can influence the node downstream of S_N via any arbitrarily large and arbitrarily interconnected embedded (sub-)network of interactions. Interactions are indicated using a solid dot, rather than an arrow or a "T", in order to indicate that the noted interactions may be either inhibitory or activating in nature. As we discuss in Supplementary Note 8, circuits should generally be negative (comprising an odd number of inhibitory interactions) in order to promote a stable steady-state. Any node could also, optionally, be regulated by node(s) from outside the module as long as those regulating nodes are "blind" regulations due to the RPA-generating activities of other parts of the network. We discuss the concept of "blind", vs "live", regulations in greater detail in Supplementary Note 5. We also note several additional optional "live" regulations in feint: any route that is contiguous with the opposer node's circuit(s) will automatically be fully opposed. Those routes may therefore also be considered part of the Module. In this context, the nodes "C" and "D" demarcate the longest route segment that is contiguous with the circuit into which the opposer node is embedded.

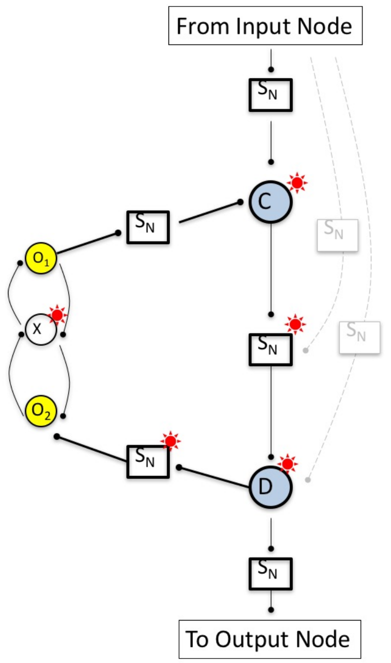

Supplementary Figure 6: General form of the Opposer Module, featuring a two-node opposing set for illustration. In common with the Opposer Module using a single opposer node (Supplementary Figure 5), the nodes "C" and "D" (both indicated in blue) delineate the feedback architecture of the module. Once again, the red superposed asterisk indicates that the associated node(s) exhibit RPA due to the "opposing" influence of the opposing set. The elements S_N once again indicate the possibility for fully embedded sub-networks at the indicated locations. The opposing set is $\{O_1, O_2\}$, while the "master set" is $\{O_1, O_2, X\}$; The family of interlinked disjoint circuits for this opposing set comprises the two circuits, (O_1, X) and (O_2, X). The complement of O_1 in the master set, for example, is the cycle (O_2, X). The complement of the cycle (O_1, X) in the master set, however, is the single node O_2, which does not represent a **circuit**. We can make a corresponding series of statements beginning with the node O_2; in this manner, it is easily seen that the opposing set $\{O_1, O_2\}$ satisfies the conditions of Theorem 3. Once again, interactions are indicated using a solid dot, rather than an arrow or a "T", in order to indicate that the noted interactions may be either inhibitory or activating in nature. As we discuss in Supplementary Note 8, circuits should generally be negative (comprising an odd number of inhibitory interactions) in order to promote a stable steady-state. Any node could also, optionally, be regulated by node(s) from outside the module as long as those regulating nodes are "blind" regulations due to the RPA-generating activities of other parts of the network. We discuss the concept of "blind", vs "live", regulations in greater detail in Supplementary Note 5. We also note several additional optional "live" regulations in feint: any route that is contiguous with the opposer node's circuit(s) will automatically be fully opposed. Those routes may therefore also be considered part of the Module. In this context, the nodes "C" and "D" demarcate the longest route segment that is contiguous with the circuit into which the opposer node is embedded.

with the steady-state thus occurring when

$$f_B^+(P_B,\{P_U\}) = f_B^-(P_B,\{P_D\}).$$

Now, suppose that there are m_u upregulators in $\{P_U\}$ and m_d downregulators in $\{P_D\}$. From the requirement of linearity among balancer steady-states, it follows that f_B must be of the general form

$$f_B = f(k_{u1}P_{u1} + k_{u2}P_{u2} + \ldots + k_{um_u}P_{um_u} + k_u + k_{ub}P_B)g(P_B)$$
$$-f(k_{d1}P_{d1} + k_{d2}P_{d2} + \ldots + k_{dm_d}P_{dm_d} + k_d + k_{db}P_B)g(P_B).$$

Thus, the elements of the forward part of the reaction, f_B^+,involving the reaction's dependency on P_B must be 'paired' with the corresponding dependency on P_B on the reverse part of the reaction, f_B^- (that is, via the function $g(P_B)$).

We also see that in order to guarantee linearity among balancer steady-states, the only allowed members of $\{P_U\}$ and $\{P_D\}$ are (i) the D-node associated to the M-set, (ii) another balancer node for that M-set, or (iii) an *adapted* node - ie. one that exhibits RPA due to the adaptive properties of other MA-subsets of the RPA equation.

By way of simple example, consider a balancer node, P_B, with a single upregulator, P_u. The most general form for reaction rate at this node would be given by the relationship

$$f_B = f_B^+(P_u, P_B) - f_B^-(P_B),$$

which, in order for P_B to function as a balancer node, would have to assume the form

$$f_B = f(k_1P_u + k_2)g(P_B) - f(k_3P_B + k_4)g(P_B).$$

An example of appropriate reaction kinetics could therefore be

$$f_B = k_1P_u - k_2P_B. \tag{39}$$

Once again, we consider the question of how such a special form could be implemented, or at least closely approximated, by established rate laws for chemical reactions. Again appealing to Michaelis-Menten reaction kinetics, Equation (39) could be approximated by

$$f_B = \frac{k_1P_u(P_{Btot} - P_B)}{K_{m1} + (P_{Btot} - P_B)} - \frac{\hat{k}_2P_B}{K_{m2} + P_B}, \tag{40}$$

where P_{Btot} represents the (fixed) sum of the active and inactive forms of P_B, and subject to the parameter constraints $K_{m1} << (P_{Btot} - P_o)$ (corresponding to the enzyme, P_u, operating *at saturation*, or *close to saturation*), and $K_{m2} >> P_B$, (corresponding to the (constant) de-activating enzyme operating *far from saturation*) with $\hat{k}_2 = k_2K_{m2}$. We see that the reaction kinetics for each of the set of balancer nodes associated to an M-set allow these balancer nodes to form a computational unit which constrains all balancer steady-states to a straight line trajectory parametrized by their associated D-node (see Theorem 5).

SN4.3 The Reaction Kinetics of Connector Nodes in M-sets

We saw in Supplementary Note 3 that the connector node, P_C associated to an M-set requires reaction kinetics that satisfy an equation of the general form,

$$\sum_i \hat{K}_i \frac{\partial f_C}{\partial P_i} = \sum_j \hat{K}_j \frac{\partial f_C}{\partial P_j},$$

for all $\mathscr{I}$ and all π_n where the indices i and j represent pre-terminal nodes (ie. immediately upstream of the C-node) for the positive and negative route segments, respectively, which in turn requires that

$$P_C^* = \hat{K}. \tag{41}$$

Whereas the reaction kinetics for both balancer nodes and opposer nodes required the mathematical role of the *substrate* to be commensurable ("paired") on both the upregulating and downregulating sides of the reaction, here we see that it is the mathematical role of the regulator nodes (the associated pre-terminal balancer nodes), not the substrate, that must be matched on both sides of the reaction rate f_C. Indeed, with the steady-states of all pre-terminal balancer nodes being linearly related to that of the D-node, as required, we see that any of the following example forms for f_C are suitable, and produce a steady-state value for P_C^* that is *adaptive* - dependent only on network parameters associated with the particular M-set:

$$f_C = \frac{k_1 P_{u1} P_{u2} (P_{Ctot} - P_C)}{K_{m1} + (P_{Ctot} - P_C)} - \frac{k_2 P_d^2 P_C}{K_{m2} + P_C}, \tag{42}$$

or

$$f_C = \frac{(k_1 P_{u1} + k_2 P_{u2})(P_{Ctot} - P_C)}{K_{m1} + (P_{Ctot} - P_C)} - \frac{k_3 P_d P_C}{K_{m2} + P_C}, \tag{43}$$

where P_{Ctot} is the (constant) sum of the active and inactive forms of P_C, and where in both cases the connector has two upregulating pre-terminal balancers, P_{u1} and P_{u2}, and one downregulating pre-terminal balancer, P_d; or

$$f_C = \frac{k_1 P_u^2 (P_{Ctot} - P_C)}{K_{m1} + (P_{Ctot} - P_C)} - \frac{k_2 P_{d1} P_{d2} P_C}{K_{m2} + P_C}, \tag{44}$$

where the connector has one upregulator and two downregulators.

It follows that in order to implement connector kinetics, a node P_C can be regulated *only* by balancer nodes (associated to the same M-set(s)) or by *adapted* nodes created by other MA-subsets. Connector nodes are themselves, by nature, adapted nodes (see Equation 41). With the upregulators and downregulators subject to balancer kinetics in Equations (42) to (44), we note that connector kinetics may be implemented by Michaelis-Menten rate laws without any constraints on parameters (eg. on the Michaelis constants K_{m1} and K_{m2}). Recall that this was not the case for opposer kinetics or balancer kinetics.

Supplementary Note 5: Independently Adapting Subsets and the Interconnectivity of Modules

In Supplementary Notes 3 and 4, we considered the solution of the RPA problem in terms of the set of all possible partitions into minimally-adaptive (MA) subsets.

Now having considered the basic mechanisms by which these MA-subsets are created, we must now confront the issue of whether or not the complement of a particular MA-subset (in $\mathscr{R}$ - the set of terms of the RPA equation) also admits a partition into MA-subsets - thereby allowing RPA to occur for the wider network as a whole. In other words, we now consdier the circumstances under which an *internally valid* MA-subset is also *relatively valid*.

To this end, we first recognise that any single mechanism (eg. the activity of an opposer node or the combined activities of a set of balancer nodes collaborating with their associated connector node) can simultaneously create multiple MA-subsets.

In particular, a single opposer node will simultaneously create S-sets from all terms in which the opposer appears as a kinetic multiplier in the cycle component of the term.

A set of balancer nodes and their associated connector node can also simultaneously create multiple M-sets. As an example of this principle, consider the simple modular configuration in Supplementary Figure 7, where the associated set of four terms is being considered as a potential M-set. The LAQ is thus $\frac{\partial f_B}{\partial A}\frac{\partial f_C}{\partial B}\frac{\partial f_D}{\partial C}\frac{\partial f_E}{\partial D} - \frac{\partial f_C}{\partial A}\frac{\partial f_D}{\partial C}\frac{\partial f_E}{\partial D}\frac{\partial f_B}{\partial B} - \frac{\partial f_B}{\partial A}\frac{\partial f_C}{\partial B}\frac{\partial f_E}{\partial C}\frac{\partial f_D}{\partial D} + \frac{\partial f_C}{\partial A}\frac{\partial f_E}{\partial C}\frac{\partial f_B}{\partial B}\frac{\partial f_D}{\partial D} = 0$. While node A is the diverter node for the M-set as a whole, and E the connector node, we observe that this selection of terms also contains an intermediate common node, C. For this reason, with C chosen to be a connector node (paired with A) and B operating as a balancer node, we see that the first two terms of the above LAQ sum to zero under this action, and that the third and fourth terms together also sum to zero independently under the same action. In this way the activity of the balancer module comprising only nodes A, B and C actually creates two M-sets. Likewise, with C chosen as a diverter node (paired with connector node E) and D assigned the role of a balancer, we see that the first and third terms together form an M-set, as do the second and fourth terms.

Thus, the ability of *any* given MA-subset-generating mechanism to simultaneously generate multiple MA-subsets, suggests that from a mathematical point of view, there exists a more fundamental partition of $\mathscr{R}$ than the partition into MA-subsets - namely the partition into *independently adapting subsets*.

For a balancing mechanism, for instance, the union of the original M-set with all other terms that are *automatically balanced* by the mechanism represents the *independently adapting subset* of $\mathscr{R}$ associated with that balancing mechanism. The general Balancer Module depicted in Figure 1c of the main paper thus represents the class of network topologies that correspond to an independently adapting (balancing) subset of an RPA equation.

Moreover, we note in the main paper that any route in a network that is only *partially* opposed must have copies that are balanced. Since a balancing mechanism will automatically balance all copies of its routes, such a partially opposed route is *redundantly opposed*. As such, the independently adapting subset associated to any opposition mechanism (via opposing sets) comprises the union of only those terms in $\mathscr{R}$ whose routes are fully opposed by the mechanism. The union of S-sets generated by partial opposition of a particular route should thus be absorbed into the independently adapting subset associated with the relevant balancing mechanism.

From the conditions of Theorems 2 and 3, then, the independently adapting subset associated with an opposition mechanism contains all copies of all routes that are disjoint from the opposing set, while contiguous with a circuit into which the opposing set is embedded - that is, all routes fully opposed by the opposition mechanism in question. The general Opposer Module presented in Figure 1(a,b) of the main paper then represents the class of network topologies that correspond to an independently adapting (opposing) subset of an RPA equation.

Now, the hallmark of an RPA-capable network is the existence of a partition of its RPA equation into independently adapting subsets. In addition, from the observation that the terms of $\mathscr{R}$ are distributed to independently adapting subsets **by route** (that is, all instances in $\mathscr{R}$ of a particular route are to be grouped together into a single such subset), it follows that these independently adapting subsets are disjoint, and

must cover $\mathscr{R}$. We have seen, moreover, that two and only two mechanisms - which we call opposition and balancing - are able to generate the independently adapting subsets of $\mathscr{R}$ in an RPA capable network, and that each such mechanism may be implemented by a rich class of sub-network topologies - opposer modules and balancer modules, respectively. Taken together, these considerations imply that a network can exhibit RPA only if it is decomposable into opposer and/or balancer modules - that is, each route for the transmission of biochemical signal from input to output must be either balanced or (fully) opposed by a single network module. These modules - Opposers and Balancers - may thus properly be called *RPA Basis Modules*.

A general RPA network could contain an arbitrary number of such modules, corresponding to its RPA equation being partitioned into (the same) arbitrary number of disjoint independently adapting subsets.

(Parenthetically, now that we have developed the concept of an *independently adapting subset* we note one small subtlety on M-sets that has been deferred until now: In Section , we analysed the conditions under which a multi-term subset of $\mathscr{R}$, comprising at least two distinct routes, could represent an M-set, and stipulated that any route present in the subset should be *fully* represented in that set. While it is possible to correctly identify the constraints on balancer and connector kinetics when one or more routes are only partially represented [2], the balancing mechanism will automatically assign all instances of those routes to the *independently adapting subset* associated with the original M-set).

We must now consider how multiple *RPA Basis Modules* can coexist in a large multi-modular network.

Now, we saw in Supplementary Note 4 that the formation of an internally-valid MA subset imposed certain conditions on the reactions kinetics of one or more nodes in order to satisfy the LAQ for that set. In particular, the ability to create an S-set required at least one *opposer node*, P_o, whose reaction kinetics satisfy $\frac{\partial f_o}{\partial P_o} = 0$ for all $\mathscr{I}$ and all π_n. Creation of an M-set requires at least one *balancer node*, P_B, where $\frac{\partial P_B^*}{\partial \hat{P}_B^*} = K_B$, and where $\hat{P}_B$ is either an *interior* node or the D-node for that M-set; creation of an M-set also requires a *connector* node, P_C, whose reaction kinetics satisfy a relation of the form $\sum K_i \frac{\partial f_C}{\partial P_i} = \sum K_j \frac{\partial f_C}{\partial P_j}$, which requires $P_C^* = \hat{K}$ (K_B and $\hat{K}$ being *constants*/parameters associated to the module).

Now, it is clear from the analysis of these three distinct modes of reaction kinetics that opposer kinetics, balancer kinetics and connector kinetics are three mutually exclusive classes of reaction kinetics. That is, a node can be *at most one* of these three special node types.

This observation points to the central criterion for determining if an internally-valid MA subset is also relatively valid: a node that plays one special role (opposer, balancer or connector node) in satisfying the LAQ for the set cannot then play a *different* special role in order to satisfy the CAQ for the set's complement in $\mathscr{R}$. We explain in the main paper that this requires the "active" part of each module (between the apex node and the base node of the module) to be distinct from the "active" part of any other module. We further explain in the paper that there are two fundamental ways for the active parts of any two modules to be distinct from each other:

1. Modules connected in parallel. In this scenario, the respective route collections for the two modules diverge upstream of the active parts of the modules, and then reconnect again downstream of the active parts. The computational nodes within the active parts of one module do not "feed into" the other module in any way.

2. Modules connected in series: In this scenario, one or more computational nodes of a module may also participate in some network route that is not opposed or balanced by that module. In other words, those computational nodes may "feed into" another module.

To speak about the series interconnections of RPA basis modules in precise topological terms, we introduce the concepts of "live" and "blind" regulations in the main paper. As we explain there, "live"

[2] albeit with the possibility that (a) redundant constraints are introduced on circuits fully disjoint from the module (since, depending on which instances of the routes are included in the proposed M-set, these circuit factors may not cancel as they would if all route instances were included), and (b) constraints on reaction kinetics may not be given directly for *all* balancer nodes by the analysis of the LAQ, in cases where circuit elements from any fully embedded circuits are not represented among the reduced collection of route instances. In both these cases, these difficulties are removed once all instances of the routes in question are incorporated into the independently adapting subset associated with the original M-set.

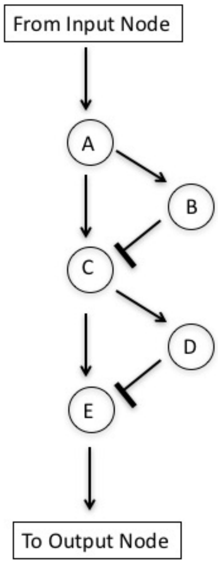

Supplementary Figure 7: A simple five-node architecture considered as an M-set containing four terms (see explanation in text). While A may be considered the diverter node, and E the connector node, for the set as a whole, we see that C is an intermediate common node between A and E. As such, either $\{A, C\}$ OR $\{C, E\}$ can operate together as a D-C node pair for the set; either way, the LAQ will be satisfied for the four-term set.

regulations are outgoing regulations from either an opposer node (or its downstream dependents) or a balancer node, since these nodes do not exhibit the RPA property. "Blind" regulations, on the other hand, come from nodes that *do* exhibit the RPA property, and include the independent regulator for an opposer node (and any of its dependents), and connector nodes (and any downstream dependents). From a mathematical point of view, in terms of the partition of $\mathscr{R}$: a blind regulation from a module only adds terms to $\mathscr{R}$ that are automatically added to the independently adapting subset associated to that module. By contrast, a live outgoing regulation introduces terms into the *complement* of the independently adapting subset for the module. For this reason, a live outgoing regulation creates the need for an ancillary module, which either balances or *fully* opposes the terms created by the interconnection. The ancillary module is thus *in series* with the original module.

In the sections to follow, we consider several concrete examples that highlight the contributions of RPA basis modules, and their interconnections, to the topologies of larger multimodular RPA-capable networks.

SN5.1 Simple examples of RPA networks comprising multiple interconnected modules

We now propose several simple examples of RPA networks which exemplify the principles we have outlined in the present study. Five different sample networks are analysed, each one comprising a different combination of RPA basis modules, selected from the two possible classes (opposer and balancer). In the explanations appended to each example, we propose the following simplified notation for the terms of the RPA equation (the set $\mathscr{R}$):

1. for a route or route-segment $\frac{\partial f_b}{\partial P_a}\frac{\partial f_c}{\partial P_b}\frac{\partial f_d}{\partial P_c}$, we write $P_a \to P_b \to P_c \to P_d$;

2. for a cycle $\frac{\partial f_b}{\partial P_a}\frac{\partial f_c}{\partial P_b}\frac{\partial f_a}{\partial P_c}$, we write $\left(P_a \to P_b \to P_c\right)$; this denotation is not unique of course - the nodes of the cycle could be written down in any cyclic permutation;

3. for a kinetic multiplier associated to a node P_a, we write $\left(P_a\right)$.

4. appended to any given term will be the double sign $(\pm)(\pm)$, where the first sign is the native sign of the term, and the second sign is the influence sign.

Thus, for a term of the RPA equation of the form $\frac{\partial f_2}{\partial P_1}\frac{\partial f_7}{\partial P_2}\frac{\partial f_3}{\partial P_5}\frac{\partial f_4}{\partial P_3}\frac{\partial f_5}{\partial P_4}\frac{\partial f_6}{\partial P_7}$ (taken from Example 2 in Supplementary Note 3), the term will appear with positive native sign. If the route component is negative, the single circuit among nodes P_3, P_4 and P_5 is negative and if the kinetic multiplier (associated with node P_6) is negative, giving a negative influence sign overall, the term will be expressed by:
$(+)(-)P_1 \to P_2 \to P_7\left(P_3 \to P_4 \to P_5\right)\left(P_6\right)$.

We now consider five different examples of RPA networks in turn.

SN5.1.1 An Opposer Module (with two-node opposing set) connected in series with another Opposer Module (with single opposer node)

A simple eight-node network is presented in Supplementary Figure 8, in which two Opposer Modules are connected in series: an upstream module with a two-node opposing set, and a downstream module with a single opposer node. For this particular network, the eight terms of the RPA equation are represented by:

$$(+)(-)A \to G \to H\left(B\right)\left(C\right)\left(D\right)\left(E\right)\left(F\right) \tag{45}$$

$$(-)(+)A \to G \to H\left(B \to C\right)\left(D\right)\left(E\right)\left(F\right) \tag{46}$$

$$(+)(-)A \to G \to H\left(B \to C\right)\left(D\right)\left(E \to F\right) \tag{47}$$

$$(+)(-)A \to G \to H\left(B\right)\left(C \to D\right)\left(E \to F\right) \tag{48}$$

$$(-)(+)A \to G \to H\left(B\right)\left(C\right)\left(D\right)\left(E \to F\right) \tag{49}$$

$$(-)(+)A \to G \to H\left(B\right)\left(C \to D\right)\left(E\right)\left(F\right) \tag{50}$$

$$(+)(-)A \to B \to C \to D \to E \to G \to H\left(F\right). \tag{51}$$

Now, we can see that nodes B and D can work together as a two-node opposing set, to fully oppose the route $A \to G \to H$ (terms 45 through 50); this requires both $\left(B\right) = \frac{\partial f_B}{\partial P_B} = 0$ and $\left(D\right) = \frac{\partial f_D}{\partial P_D} = 0$. Both B and D participate in the route $A \to B \to C \to D \to E \to G \to H$, which corresponds to a "live" outgoing regulation from the upstream module. That route (term 51) may be opposed by the node F, however, with $\left(F\right) = \frac{\partial f_F}{\partial P_F} = 0$. Thus, the output node, H, will exhibit RPA under the influence of varying inputs delivered to the input node, A, since it consists of an Opposer Module (using the two-node opposing set $\{B, D\}$), connected in series with the downstream Opposer Module (with opposer node F). Simulations of this network are displayed in Supplementary Figure 8, using the simple reaction equations given below (which conform to the requisite opposer kinetics for nodes B, D and F). Parameters are given in the caption to the

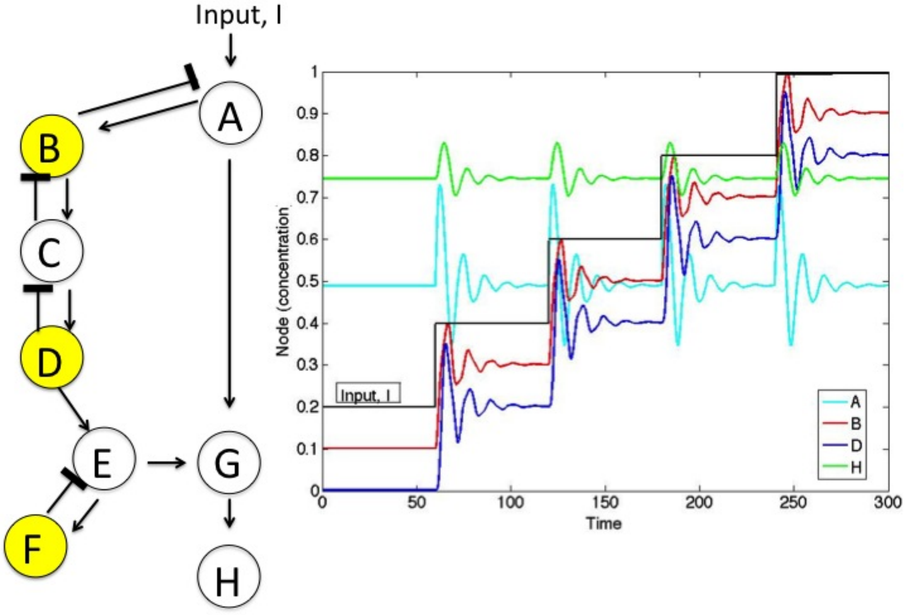

Supplementary Figure 8: A simple eight-node network configuration in which an opposer module (with two-node opposing set, upstream) is connected in series with another opposer module (with single opposer node, downstream). The opposing set for the upstream Opposer Module is $\{B, D\}$, the corresponding master set being $\{B, C, D\}$; the route $A \to G \to H$ is fully opposed by this opposing set. The only route that is not opposed by the set is the route in which those opposer nodes actually participate, which in this example is the route $A \to B \to C \to D \to E \to G \to H$. That route is fully opposed by the single opposer node, F, however, since this is disjoint from the route and participates in one circuit which is contiguous with the route. Time-dependent solutions to the modeling equations (45) through (51) are shown on the right-hand side of the figure for several nodes. The input stimulus, indicated in black begins at 0.2 and increases in steps of 0.2 until a value of unity. As shown, the output node (H) exhibits perfect robust adaptation, as do nodes C, E, G and A (not shown). Nodes B and D, being opposer nodes, do not adapt as shown. Node F, which is a (single) opposer similarly does not adapt (not shown). Parameters for the modeling equations (45) through (51) are $k_1 = 1, k_2 = 0.2, k_3 = 1, k_4 = 0.5, k_5 = 0.5, k_6 = 1, k_7 = 1, k_8 = 0.2, k_9 = 1, k_{10} = 0.5, k_{11} = 0.1, k_{12} = 0.1, k_{13} = 5, k_{14} = 1, k_{15} = 0.5, k_{16} = 1, k_{17} = 2, k_{18} = 0.5, k_{19} = 0.5, k_{20} = 2$.

figure.

$$f_A = \frac{dA}{dt} = k_1 I - k_2 A - k_3 B,$$
$$f_B = \frac{dB}{dt} = k_4 A - k_5 C,$$
$$f_C = \frac{dC}{dt} = k_6 B - k_7 D - k_8 C,$$
$$f_D = \frac{dD}{dt} = k_9 C - k_{10},$$
$$f_E = \frac{dE}{dt} = k_{11} D - k_{12} E - k_{13} F,$$
$$f_F = \frac{dF}{dt} = k_{14} E - k_{15},$$
$$f_G = \frac{dG}{dt} = k_{16} A + k_{17} E - k_{18} G,$$
$$f_H = \frac{dH}{dt} = k_{19} G - k_{20} H.$$

SN5.1.2 An Opposer Module connected in series with a Balancer Module

A thirteen-node network is presented in Supplementary Figure 9, in which an Opposer Module (with a single opposer node) is connected in series with a Balancer Module (comprising five balancer nodes). The terms of the RPA equation for this network are:

$$(-)(-)P_1 \to P_2 \to P_3 \to P_{11} \to P_{12} \to P_{13}\Big(P_4\Big)\Big(P_5\Big)\Big(P_6\Big)\Big(P_7\Big)\Big(P_8\Big)\Big(P_9\Big)\Big(P_{10}\Big) \quad (52)$$

$$(+)(+)P_1 \to P_9 \to P_{11} \to P_{12} \to P_{13}\Big(P_2\Big)\Big(P_3\Big)\Big(P_4\Big)\Big(P_5\Big)\Big(P_6\Big)\Big(P_7\Big)\Big(P_8\Big)\Big(P_{10}\Big) \quad (53)$$

$$(-)(-)P_1 \to P_9 \to P_{11} \to P_{10} \to P_3 \to P_4 \to P_5 \to P_{13}\Big(P_2\Big)\Big(P_6\Big)\Big(P_7\Big)\Big(P_8\Big)\Big(P_{12}\Big) \quad (54)$$

$$(+)(-)P_1 \to P_9 \to P_{11} \to P_{10} \to P_3 \to P_6 \to P_7 \to P_8 \to P_{13}\Big(P_2\Big)\Big(P_4\Big)\Big(P_5\Big)\Big(P_{12}\Big) \quad (55)$$

$$(-)(-)P_1 \to P_2 \to P_3 \to P_4 \to P_5 \to P_{13}\Big(P_6\Big)\Big(P_7\Big)\Big(P_8\Big)\Big(P_9\Big)\Big(P_{10}\Big)\Big(P_{11}\Big)\Big(P_{12}\Big) \quad (56)$$

$$(+)(-)P_1 \to P_2 \to P_3 \to P_6 \to P_7 \to P_8 \to P_{13}\Big(P_4\Big)\Big(P_5\Big)\Big(P_9\Big)\Big(P_{10}\Big)\Big(P_{11}\Big)\Big(P_{12}\Big) \quad (57)$$

We see that the single node P_{10} occurs in a circuit and also appears in the routes incorporating the segment $P_1 \to P_9 \to P_{11} \to P_{10} \to P_3$. For all *other* routes, P_{10}'s only circuit is contiguous. It therefore fully opposes all other such routes, and P_{10} creates S-sets from terms (52), (53), (56) and (57). This leaves terms (54) and (55), containing the routes in which P_{10} appears, which may both be assigned to a single M-set since their net signs are opposite, and since the associated interior nodes and C-node do not include P_{10}. Indeed, the rLAQ for this M-set is

$$\left|\frac{\partial f_4}{\partial P_3}\frac{\partial f_5}{\partial P_4}\frac{\partial f_{13}}{\partial P_5}\frac{\partial f_6}{\partial P_6}\frac{\partial f_7}{\partial P_7}\frac{\partial f_8}{\partial P_8}\right| = \left|\frac{\partial f_6}{\partial P_3}\frac{\partial f_7}{\partial P_6}\frac{\partial f_8}{\partial P_7}\frac{\partial f_{13}}{\partial P_8}\frac{\partial f_4}{\partial P_4}\frac{\partial f_5}{\partial P_5}\right|,$$

or, dividing through by common kinetic multipliers,

$$\left|\frac{\frac{\partial f_4}{\partial P_3}\frac{\partial f_5}{\partial P_4}}{\frac{\partial f_4}{\partial P_4}\frac{\partial f_5}{\partial P_5}}\right|\frac{\partial f_{13}}{\partial P_5} = \left|\frac{\frac{\partial f_6}{\partial P_3}\frac{\partial f_7}{\partial P_6}\frac{\partial f_8}{\partial P_7}}{\frac{\partial f_6}{\partial P_6}\frac{\partial f_7}{\partial P_7}\frac{\partial f_8}{\partial P_8}}\right|\left|\frac{\partial f_{13}}{\partial P_8}\right|.$$

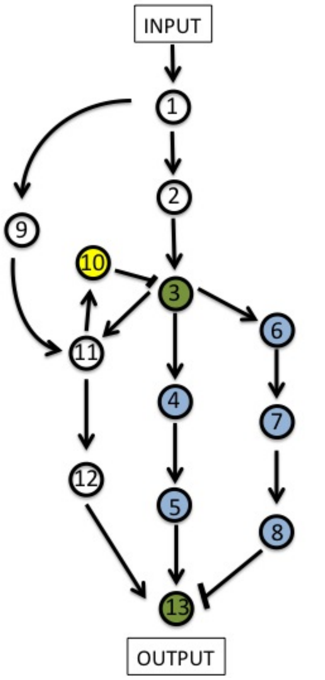

Supplementary Figure 9: A network schematic for a thirteen-node network comprising an Opposer Module (upstream) connected in series with a Balancer Module (downstream). The single opposer node, P_{10} is indicated in yellow; the C-D node pair for the balancer module, P_3 and P_{13}, are indicated in green, with the corresponding balancer nodes, P_4, P_5, P_6, P_7 and P_8 indicated in blue.

As explained in Supplementary Note 3, this requires:

$$\frac{dP_4^*}{dP_3^*} = K_{43},$$
$$\frac{dP_5^*}{dP_4^*} = K_{54},$$
$$\frac{dP_6^*}{dP_3^*} = K_{63},$$
$$\frac{dP_7^*}{dP_6^*} = K_{76},$$
$$\frac{dP_8^*}{dP_7^*} = K_{87},$$

where $K_{43}, K_{54}, K_{63}, K_{76}$ and K_{87} are constants that are independent of the steady-states of the nodes appearing in the rLAQ. Then, f_{13} must satisfy

$$\kappa_5 \frac{\partial f_{13}}{\partial P_5} = \kappa_8 \left| \frac{\partial f_{13}}{\partial P_8} \right|,$$

where $\kappa_5 = |K_{43}K_{54}|$ and $\kappa_8 = |K_{54}K_{76}K_{87}|$.

Simulations of this network are displayed in Supplementary Figure 10, using the following equations

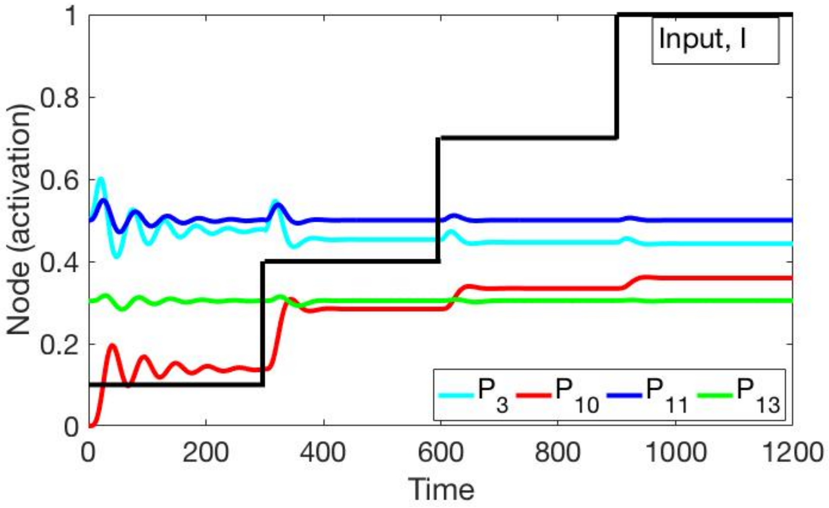

Supplementary Figure 10: Time-dependent solutions for the modeling equations given in Equations (58) through (70). Note that the only Equations in this set that must conform to a specific form are Equations (61) through (65), which require balancer kinetics, Equation (70) which requires connector kinetics, and Equation (67) which requires opposer kinetics. All other equations could assume any functional form that encodes the nodal regulations noted in Supplementary Figure 9. Moreover, the specific forms for opposer, balancer and connector kinetics chosen here are only simple examples for illustrative purposes. The input stimulus, indicated in black, is a step function that begins at 0.1 and increases in increments of 0.3 until a value of unity. As shown, the output node, P_{13}, as well as P_{11} (which regulates the opposer node P_{10}) exhibit RPA in response to the varying input. Node P_{12} (not shown) also exhibits RPA. On the other hand, the opposer node, P_{10} and the connector node, P_3 do not adapt, as shown. Nodes P_4, P_5, P_6, P_7 and P_8, being balancer nodes, do not adapt (not shown), nor do nodes P_1, P_2, P_3, or P_9 (not shown). Parameters for Equations (58) through (70) are: $k_1 = 0.2, k_2 = 0.1, k_3 = 0.2, k_4 = 0.1, k_5 = 0.2, k_6 = 0.4, k_7 = 0.2, k_8 = 0.1, k_9 = 0.2, k_{10} = 0.1, k_{11} = 0.3, k_{12} = 0.1, k_{13} = 0.2, k_{14} = 0.1, k_{15} = 0.3, k_{16} = 0.1, k_{17} = 0.2, k_{18} = 0.1, k_{19} = 0.2, k_{20} = 0.2, k_{21} = 0.2, k_{22} = 0.1, k_{23} = 0.1, k_{24} = 0.2, k_{25} = 0.1, k_{26} = 0.2, k_{27} = 0.1, k_{28} = 0.1, P_{1tot} = P_{2tot} = P_{3tot} = P_{4tot} = P_{5tot} = P_{6tot} = P_{7tot} = P_{8tot} = P_{9tot} = P_{10tot} = P_{11tot} = P_{12tot} = P_{13tot} = 1$.

(with parameters as given in the figure caption):

$$f_1 = \frac{dP_1}{dt} = k_1 I(P_{1tot} - P_1) - k_2 P_1, \tag{58}$$

$$f_2 = \frac{dP_2}{dt} = k_3 P_1 (P_{2tot} - P_2) - k_4 P_2, \tag{59}$$

$$f_3 = \frac{dP_3}{dt} = k_5 P_2 (P_{3tot} - P_3) - k_6 P_3 P_{10}, \tag{60}$$

$$f_4 = \frac{dP_4}{dt} = k_7 P_3 - k_8 P_4, \tag{61}$$

$$f_5 = \frac{dP_5}{dt} = k_9 P_4 - k_{10} P_5, \tag{62}$$

$$f_6 = \frac{dP_6}{dt} = k_{11} P_3 - k_{12} P_6, \tag{63}$$

$$f_7 = \frac{dP_7}{dt} = k_{13} P_6 - k_{14} P_7, \tag{64}$$

$$f_8 = \frac{dP_8}{dt} = k_{15} P_7 - k_{16} P_8, \tag{65}$$

$$f_9 = \frac{dP_9}{dt} = k_{17} P_1 (P_{9tot} - P_9) - k_{19} P_9, \tag{66}$$

$$f_{10} = \frac{dP_{10}}{dt} = k_{19} P_{11} - k_{20}, \tag{67}$$

$$f_{11} = \frac{dP_{11}}{dt} = k_{21}(P_3 + k_{22} P_9)(P_{11tot} - P_{11}) - k_{23} P_{11}, \tag{68}$$

$$f_{12} = \frac{dP_{12}}{dt} = k_{24} P_{11} (P_{12tot} - P_{12}) - k_{25} P_{12}, \tag{69}$$

$$f_{13} = \frac{dP_{13}}{dt} = k_{26}(P_5 + k_{27} P_{12}) - k_{28} P_8 P_{13}. \tag{70}$$

SN5.1.3 Two Different Solutions for a Single Twelve-Node Network: A single Opposer Module, or an Opposer Module connected in series with a Balancer Module

Supplementary Figures 11 and 12 depict two different possible solutions to the RPA problem for a single network architecture comprising twelve interacting nodes: a single Opposer Module (Supplementary Figure 11), or, alternatively, an Opposer Module connected in series with a balancer module (Supplementary Figure 12). In either case, the RPA equation comprises five terms, being:

$$(+)(-)P_1 \to P_2 \to P_3 \to P_5 \to P_{12}\Big(P_4\Big)\Big(P_6\Big)\Big(P_7\Big)\Big(P_8\Big)\Big(P_9\Big)\Big(P_{10}\Big)\Big(P_{11}\Big) \tag{71}$$

$$(+)(-)P_1 \to P_2 \to P_4 \to P_5 \to P_{12}\Big(P_3\Big)\Big(P_6\Big)\Big(P_7\Big)\Big(P_8\Big)\Big(P_9\Big)\Big(P_{10}\Big)\Big(P_{11}\Big) \tag{72}$$

$$(-)(+)P_1 \to P_2 \to P_6 \to P_8 \to P_{11} \to P_{12}\Big(P_3\Big)\Big(P_4\Big)\Big(P_5\Big)\Big(P_7\Big)\Big(P_9\Big)\Big(P_{10}\Big) \tag{73}$$

$$(+)(-)P_1 \to P_2 \to P_6 \to P_8 \to P_{10} \to P_{11} \to P_{12}\Big(P_3\Big)\Big(P_4\Big)\Big(P_5\Big)\Big(P_7\Big)\Big(P_9\Big) \tag{74}$$

$$(+)(+)P_1 \to P_2 \to P_6 \to P_8 \to P_9 \to P_{11} \to P_{12}\Big(P_3\Big)\Big(P_4\Big)\Big(P_5\Big)\Big(P_7\Big)\Big(P_{10}\Big) \tag{75}$$

Now, it is clear that the node P_7 is disjoint from all routes of the network, and participates in a circuit that is contiguous with all routes of the network. Thus, P_7 may act as a single opposer for the entire network. Simulations for this RPA solution (corresponding to the schematic in Supplementary Figure 11) are given in Supplementary Figure 13, using the following set of equations, where the node P_7 operates with the

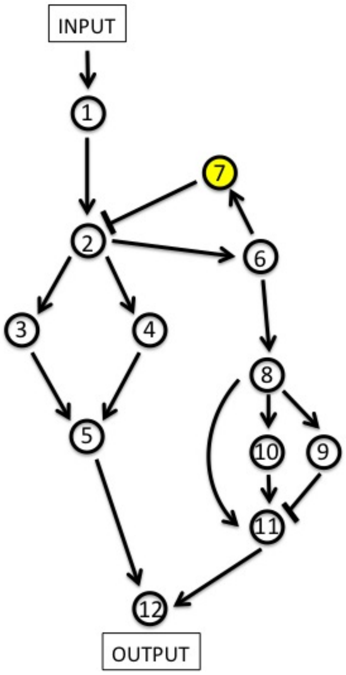

Supplementary Figure 11: A network schematic for a set of twelve interconnected nodes, showing one possible solution to the RPA equation for this particular arrangement of nodes. In particular, the node P_7 acts as an opposer node, and fully opposes *all* routes of this network. The network thus comprises a single Opposer Module.

requisite opposer kinetics:

$$f_1 = \frac{dP_1}{dt} = k_1 I(P_{1tot} - P_1) - k_2 P_1, \tag{76}$$

$$f_2 = \frac{dP_2}{dt} = k_3 P_1(P_{2tot} - P_2) - k_4 P_2 P_7, \tag{77}$$

$$f_3 = \frac{dP_3}{dt} = k_5 P_2(P_{3tot} - P_3) - k_6 P_3, \tag{78}$$

$$f_4 = \frac{dP_4}{dt} = k_7 P_2(P_{4tot} - P_4) - k_8 P_4, \tag{79}$$

$$f_5 = \frac{dP_5}{dt} = k_9(P_3 + k_{10} P_4)(P_{5tot} - P_5) - k_{11} P_5, \tag{80}$$

$$f_6 = \frac{dP_6}{dt} = k_{12} P_2(P_{6tot} - P_6) - k_{13} P_6, \tag{81}$$

$$f_7 = \frac{dP_7}{dt} = k_{14} P_6 - k_{15}, \tag{82}$$

$$f_8 = \frac{dP_8}{dt} = k_{16} P_6(P_{8tot} - P_8) - k_{17} P_8, \tag{83}$$

$$f_9 = \frac{dP_9}{dt} = k_{18} P_8 - k_{19} P_9, \tag{84}$$

$$f_{10} = \frac{dP_{10}}{dt} = k_{20} P_8 - k_{21} P_{10}, \tag{85}$$

$$f_{11} = \frac{dP_{11}}{dt} = k_{22}(P_8 + k_{23} P_{10})(P_{11tot} - P_{11}) - k_{24} P_{11} P_9, \tag{86}$$

$$f_{12} = \frac{dP_{12}}{dt} = k_{25}(P_{11} + k_{26} P_5)(P_{12tot} - P_{12}) - k_{27} P_{12}. \tag{87}$$

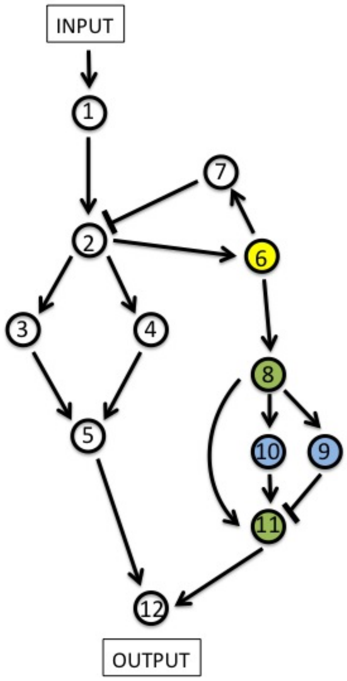

Supplementary Figure 12: The same interconnectivity of nodes as in Supplementary Figure 11 is displayed here, but with a different solution to the RPA problem. Here the node P_6 acts as a single opposer node, and thus fully opposes the routes $P_1 \rightarrow P_2 \rightarrow P_3 \rightarrow P_5 \rightarrow P_{12}$ and $P_1 \rightarrow P_2 \rightarrow P_4 \rightarrow P_5 \rightarrow P_{12}$. The routes that opposer P_6 participates in, however, are "balanced" by the balancer module with diverter node P_8, connector node P_{11} and balancer nodes P_9 and P_{10}.

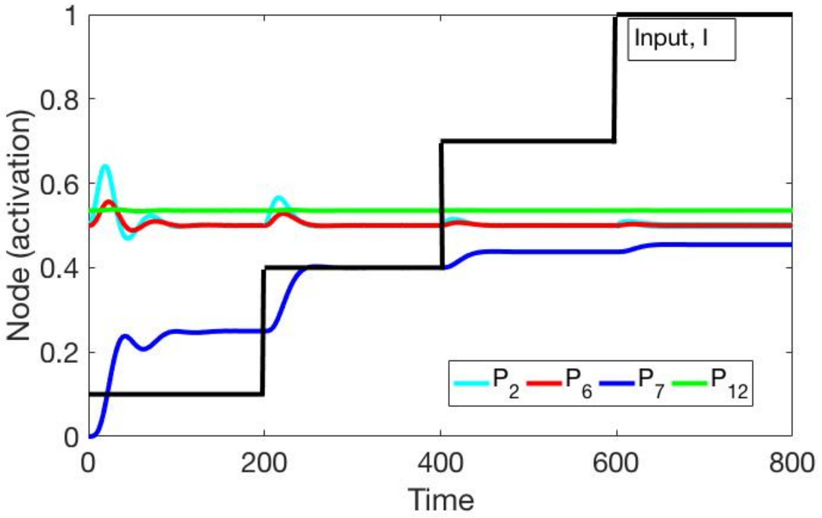

Supplementary Figure 13: Time-dependent solutions to the modeling equations (76) through (87). Here, only the opposer node, P_7, has constraints on viable reaction kinetics (ie. it must assume opposer kinetics); all other reactions are unconstrained. The input, I, indicated in black, is a step function beginning at a value of 0.1 and increasing in increments of 0.3 until a value of 1. For this particular solution to the RPA problem, *all* nodes exhibit RPA in response to the varying stimulus, except P_7, the opposer node, and P_1, the input node. Nodes P_2, P_6, P_7 (the opposer) and P_{13} (the output) are displayed as a representative sample. Parameters for the modeling equations are: $k_1 = 0.2, k_2 = 0.02, k_3 = 0.2, k_4 = 0.4, k_5 = 0.2, k_6 = 0.1, k_7 = 0.2, k_8 = 0.1, k_9 = 0.2, k_{10} = 0.1, k_{11} = 0.1, k_{12} = 0.2, k_{13} = 0.1, k_{14} = 0.2, k_{15} = 0.1, k_{16} = 0.2, k_{17} = 0.1, k_{18} = 0.2, k_{19} = 0.1, k_{20} = 0.1, k_{21} = 0.1, k_{22} = 0.2, k_{23} = 0.1, k_{24} = 0.1, k_{25} = 0.2, k_{26} = 0.1, k_{27} = 0.1, P_{1tot} = P_{2tot} = P_{3tot} = P_{4tot} = P_{5tot} = P_{6tot} = P_{7tot} = P_{8tot} = P_{9tot} = P_{10tot} = P_{11tot} = P_{12tot} = 1$.

Alternatively, we can obtain a different solution to the RPA problem for this network by observing that the node P_6, when endowed with opposer kinetics, creates S-sets from the terms (71) and (72). The remaining terms of the RPA equation ((73) through (75)) can then be assigned to a single M-set with rLAQ:

$$\left|\frac{\partial f_{11}}{\partial P_8}\frac{\partial f_9}{\partial P_9}\frac{\partial f_{10}}{\partial P_{10}}\right| + \left|\frac{\partial f_{10}}{\partial P_8}\frac{\partial f_{11}}{\partial P_{10}}\frac{\partial f_9}{\partial P_9}\right| = \left|\frac{\partial f_9}{\partial P_8}\frac{\partial f_{11}}{\partial P_9}\frac{\partial f_{10}}{\partial P_{10}}\right|,$$

or,

$$\frac{\partial f_{11}}{\partial P_8} + \left|\frac{\frac{\partial f_{10}}{\partial P_8}}{\frac{\partial f_{10}}{\partial P_{10}}}\right|\frac{\partial f_{11}}{\partial P_8} = \left|\frac{\frac{\partial f_9}{\partial P_8}}{\frac{\partial f_9}{\partial P_9}}\right|\left|\frac{\partial f_{11}}{\partial P_9}\right|,$$

requiring

$$\frac{dP_{10}^*}{dP_8^*} = K_{108},$$
$$\frac{dP_9^*}{dP_8^*} = K_{98},$$

with f_{10} then assuming a form which permits

$$P_{11}^* = K_{11},$$

where K_{11} is a constant depending only on kinetic parameters, and not on the steady-states of the nodes of the M-set. We observe that in the set of modeling equations given above for the single-opposer solution to

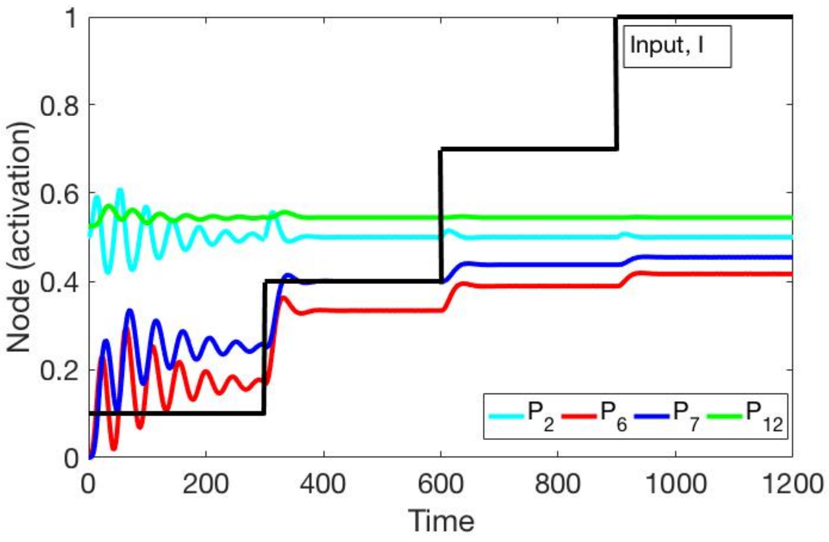

Supplementary Figure 14: Time-dependent solutions for the modeling equations (76) through (87), but with Equations (88) and (89) substituted for Equations (81) and (82) in the original set. Parameters are identical as in the previous case (see caption to Supplementary Figure 13).

this network, the reactions f_9, f_{10} and f_{11} are already in the requisite forms for balancer nodes P_8 and P_9, and connector node P_{10} (although these reactions could have assumed *any* form for the single-opposer solution, as they were 'unconstrained' nodes in that case). It therefore only remains to modify reactions f_6 and f_7 to allow P_6 to now act as an opposer, and P_7 to no longer act as an opposer, for example

$$f_6 = \frac{dP_6}{dt} = k_6 P_2 - k_{13}, \tag{88}$$

$$f_7 = \frac{dP_7}{dt} = k_{14} P_6 (P_{7tot} - P_7) - k_{15} P_7. \tag{89}$$

With these two equations substituted for the f_6 and f_7 in the original set, we show the simulations for this alternative RPA solution in Supplementary Figure 14 (with parameters as given in the figure caption).

SN5.1.4 Two Opposer Modules connected in Parallel

Supplementary Figure 15 gives a schematic of a twenty-node network architecture that comprises a large number of routes in comparison with the previous examples. Indeed, this particular network has a twenty-

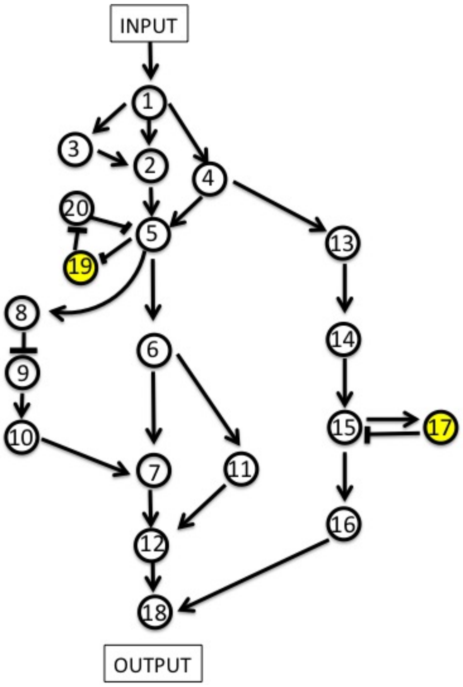

Supplementary Figure 15: Schematic representation of an twenty-node network in which two Opposer Modules (each using a single opposer node) are connected in parallel to allow the network to exhibit RPA.

term RPA equation represented by

$$(+)(-)P_1 \to P_2 \to P_5 \to P_6 \to P_7 \to P_{12} \to P_{18}\left(P_3\right)\left(P_4\right)\left(P_8\right)\left(P_9\right)\left(P_{10}\right)\left(P_{11}\right)\left(P_{13}\right)\left(P_{14}\right)\left(P_{15}\right)\left(P_{16}\right)\left(P_{17}\right)\left(P_{19}\right)\left(P_{20}\right) \tag{90}$$

$$(-)(+)P_1 \to P_2 \to P_5 \to P_6 \to P_7 \to P_{12} \to P_{18}\left(P_3\right)\left(P_4\right)\left(P_8\right)\left(P_9\right)\left(P_{10}\right)\left(P_{11}\right)\left(P_{13}\right)\left(P_{14}\right)\left(P_{15} \to P_{17}\right)\left(P_{16}\right)\left(P_{19}\right)\left(P_{20}\right) \tag{91}$$

$$(-)(+)P_1 \to P_3 \to P_2 \to P_5 \to P_6 \to P_7 \to P_{12} \to P_{18}\left(P_4\right)\left(P_8\right)\left(P_9\right)\left(P_{10}\right)\left(P_{11}\right)\left(P_{13}\right)\left(P_{14}\right)\left(P_{15}\right)\left(P_{16}\right)\left(P_{17}\right)\left(P_{19}\right)\left(P_{20}\right) \tag{92}$$

$$(+)(-)P_1 \to P_3 \to P_2 \to P_5 \to P_6 \to P_7 \to P_{12} \to P_{18}\left(P_4\right)\left(P_8\right)\left(P_9\right)\left(P_{10}\right)\left(P_{11}\right)\left(P_{13}\right)\left(P_{14}\right)\left(P_{15} \to P_{17}\right)\left(P_{16}\right)\left(P_{19}\right)\left(P_{20}\right) \tag{93}$$

$$(+)(-)P_1 \to P_4 \to P_5 \to P_6 \to P_7 \to P_{12} \to P_{18}\left(2\right)\left(P_3\right)\left(P_8\right)\left(P_9\right)\left(P_{10}\right)\left(P_{11}\right)\left(P_{13}\right)\left(P_{14}\right)\left(P_{15}\right)\left(P_{16}\right)\left(P_{17}\right)\left(P_{19}\right)\left(P_{20}\right) \tag{94}$$

$$(-)(+)P_1 \to P_4 \to P_5 \to P_6 \to P_7 \to P_{12} \to P_{18}\left(2\right)\left(P_3\right)\left(P_8\right)\left(P_9\right)\left(P_{10}\right)\left(P_{11}\right)\left(P_{13}\right)\left(P_{14}\right)\left(P_{15} \to P_{17}\right)\left(P_{16}\right)\left(P_{19}\right)\left(P_{20}\right) \tag{95}$$

$$(+)(+)P_1 \to P_2 \to P_5 \to P_8 \to P_9 \to P_{10} \to P_7 \to P_{12} \to P_{18}\left(P_3\right)\left(P_4\right)\left(P_6\right)\left(P_{11}\right)\left(P_{13}\right)\left(P_{14}\right)\left(P_{15}\right)\left(P_{16}\right)\left(P_{17}\right)\left(P_{19}\right)\left(P_{20}\right) \tag{96}$$

$$(-)(-)P_1 \to P_2 \to P_5 \to P_8 \to P_9 \to P_{10} \to P_7 \to P_{12} \to P_{18}\left(P_3\right)\left(P_4\right)\left(P_6\right)\left(P_{11}\right)\left(P_{13}\right)\left(P_{14}\right)\left(P_{15} \to P_{17}\right)\left(P_{16}\right)\left(P_{19}\right)\left(P_{20}\right) \tag{97}$$

$$(-)(-)P_1 \to P_3 \to P_2 \to P_5 \to P_8 \to P_9 \to P_{10} \to P_7 \to P_{12} \to P_{18}\left(P_4\right)\left(P_6\right)\left(P_{11}\right)\left(P_{13}\right)\left(P_{14}\right)\left(P_{15}\right)\left(P_{16}\right)\left(P_{17}\right)\left(P_{19}\right)\left(P_{20}\right) \tag{98}$$

$$(+)(+)P_1 \to P_3 \to P_2 \to P_5 \to P_8 \to P_9 \to P_{10} \to P_7 \to P_{12} \to P_{18}\Big(P_4\Big)\Big(P_6\Big)\Big(P_{11}\Big)\Big(P_{13}\Big)\Big(P_{14}\Big)\Big(P_{15} \to P_{17}\Big)\Big(P_{16}\Big)\Big(P_{19}\Big)\Big(P_{20}\Big) \tag{99}$$

$$(+)(+)P_1 \to P_4 \to P_5 \to P_8 \to P_9 \to P_{10} \to P_7 \to P_{12} \to P_{18}\Big(P_2\Big)\Big(P_3\Big)\Big(P_6\Big)\Big(P_{11}\Big)\Big(P_{13}\Big)\Big(P_{14}\Big)\Big(P_{15}\Big)\Big(P_{16}\Big)\Big(P_{17}\Big)\Big(P_{19}\Big)\Big(P_{20}\Big) \tag{100}$$

$$(-)(-)P_1 \to P_4 \to P_5 \to P_8 \to P_9 \to P_{10} \to P_7 \to P_{12} \to P_{18}\Big(P_2\Big)\Big(P_3\Big)\Big(P_6\Big)\Big(P_{11}\Big)\Big(P_{13}\Big)\Big(P_{14}\Big)\Big(P_{15} \to P_{17}\Big)\Big(P_{16}\Big)\Big(P_{19}\Big)\Big(P_{20}\Big) \tag{101}$$

$$(+)(-)P_1 \to P_2 \to P_5 \to P_6 \to P_{11} \to P_{12} \to P_{18}\Big(P_3\Big)\Big(P_4\Big)\Big(P_7\Big)\Big(P_8\Big)\Big(P_9\Big)\Big(P_{10}\Big)\Big(P_{13}\Big)\Big(P_{14}\Big)\Big(P_{15}\Big)\Big(P_{16}\Big)\Big(P_{17}\Big)\Big(P_{19}\Big)\Big(P_{20}\Big) \tag{102}$$

$$(-)(+)P_1 \to P_2 \to P_5 \to P_6 \to P_{11} \to P_{12} \to P_{18}\Big(P_3\Big)\Big(P_4\Big)\Big(P_7\Big)\Big(P_8\Big)\Big(P_9\Big)\Big(P_{10}\Big)\Big(P_{13}\Big)\Big(P_{14}\Big)\Big(P_{15} \to P_{17}\Big)\Big(P_{16}\Big)\Big(P_{19}\Big)\Big(P_{20}\Big) \tag{103}$$

$$(-)(+)P_1 \to P_3 \to P_2 \to P_5 \to P_6 \to P_{11} \to P_{12} \to P_{18}\Big(P_4\Big)\Big(P_7\Big)\Big(P_8\Big)\Big(P_9\Big)\Big(P_{10}\Big)\Big(P_{13}\Big)\Big(P_{14}\Big)\Big(P_{15}\Big)\Big(P_{16}\Big)\Big(P_{17}\Big)\Big(P_{19}\Big)\Big(P_{20}\Big) \tag{104}$$

$$(+)(-)P_1 \to P_3 \to P_2 \to P_5 \to P_6 \to P_{11} \to P_{12} \to P_{18}\Big(P_4\Big)\Big(P_7\Big)\Big(P_8\Big)\Big(P_9\Big)\Big(P_{10}\Big)\Big(P_{13}\Big)\Big(P_{14}\Big)\Big(P_{15} \to P_{17}\Big)\Big(P_{16}\Big)\Big(P_{19}\Big)\Big(P_{20}\Big) \tag{105}$$

$$(+)(-)P_1 \to P_4 \to P_5 \to P_6 \to P_{11} \to P_{12} \to P_{18}\Big(2\Big)\Big(P_3\Big)\Big(P_7\Big)\Big(P_8\Big)\Big(P_9\Big)\Big(P_{10}\Big)\Big(P_{13}\Big)\Big(P_{14}\Big)\Big(P_{15}\Big)\Big(P_{16}\Big)\Big(P_{17}\Big)\Big(P_{19}\Big)\Big(P_{20}\Big) \tag{106}$$

$$(-)(+)P_1 \to P_4 \to P_5 \to P_6 \to P_{11} \to P_{12} \to P_{18}\Big(2\Big)\Big(P_3\Big)\Big(P_7\Big)\Big(P_8\Big)\Big(P_9\Big)\Big(P_{10}\Big)\Big(P_{13}\Big)\Big(P_{14}\Big)\Big(P_{15} \to P_{17}\Big)\Big(P_{16}\Big)\Big(P_{19}\Big)\Big(P_{20}\Big) \tag{107}$$

$$(+)(-)P_1 \to P_4 \to P_{13} \to P_{14} \to P_{15} \to P_{16} \to P_{18}\Big(P_2\Big)\Big(P_3\Big)\Big(P_5\Big)\Big(P_6\Big)\Big(P_7\Big)\Big(P_8\Big)\Big(P_9\Big)\Big(P_{10}\Big)\Big(P_{11}\Big)\Big(P_{12}\Big)\Big(P_{17}\Big)\Big(P_{19}\Big)\Big(P_{20}\Big) \tag{108}$$

$$(-)(+)P_1 \to P_4 \to P_{13} \to P_{14} \to P_{15} \to P_{16} \to P_{18}\Big(P_2\Big)\Big(P_3\Big)\Big(P_6\Big)\Big(P_7\Big)\Big(P_8\Big)\Big(P_9\Big)\Big(P_{10}\Big)\Big(P_{11}\Big)\Big(P_{12}\Big)\Big(P_{17}\Big)\Big(P_5 \to P_{19} \to P_{20}\Big) \tag{109}$$

We thus see that several different partitions into *independently adapting subsets* are possible for this partcular network configuration. We see that P_{17} is disjoint from all routes of the network, and participates in a circuit that is contiguous with the route $P_1 \to P_4 \to P_{13} \to P_{14} \to P_{15} \to P_{16} \to P_{18}$, so that P_{17} (when equipped with the requisite reaction kinetics) is able to act as an opposer for that route, creating S-sets from the last two terms. The remaining terms could be assigned to a single M-set, for example, assigning P_5 the role of the D-node, and P_{12} the role of the C-node, with interior nodes being $P_6, P_7, P_8, P_9, P_{10}$ and P_{11}. For illustrative purposes, we depict a further possibility, noting that nodes P_{19} and P_{20} are disjoint from all routes, and participate in a circuit that is contiguous with all routes other than $P_1 \to P_4 \to P_{13} \to P_{14} \to P_{15} \to P_{16} \to P_{18}$ (which can be opposerd by P_{17}, as outlined above. Thus, either P_{19} or P_{20} could create S-sets from all remaining terms when equipped with suitable opposer kinetics. Here, P_{19} is chosen as the opposer node for illustrative purposes. Thus, the network as a whole may be considered as two Opposer Modules (one using P_{17} as a single opposer node, and the other using P_{19} as a single opposer node), connected together in parallel. Simulations of this solution to the RPA equation are presented in

Supplementary Figure 16, using the following equations (and parameters as given in the figure caption):

$$f_1 = \frac{dP_1}{dt} = k_1(P_{1tot} - P_1)I - k_2P_1, \tag{110}$$

$$f_2 = \frac{dP_2}{dt} = k_3(P_{2tot} - P_2)(P_1 + k_4P_3) - k_5P_2, \tag{111}$$

$$f_3 = \frac{dP_3}{dt} = k_6(P_{3tot} - P_3)P_1 - k_7P_3, \tag{112}$$

$$f_4 = \frac{dP_4}{dt} = k_8(P_{4tot} - P_4)P_1 - k_9P_4, \tag{113}$$

$$f_5 = \frac{dP_5}{dt} = k_{10}(P_{5tot} - P_5)(P_2 + k_{11}P_4) - k_{12}P_5P_{20}, \tag{114}$$

$$f_6 = \frac{dP_6}{dt} = k_{13}(P_{6tot} - P_6)P_5 - k_{14}P_6, \tag{115}$$

$$f_7 = \frac{dP_7}{dt} = k_{15}(P_6 + k_{16}P_{10})(P_{7tot} - P_7) - k_{17}P_7, \tag{116}$$

$$f_8 = \frac{dP_8}{dt} = k_{18}P_5(P_{8tot} - P_8) - k_{19}P_8, \tag{117}$$

$$f_9 = \frac{dP_9}{dt} = k_{20}(P_{9tot} - P_9) - k_{21}(P_9 + k_{21a}P_8), \tag{118}$$

$$f_{10} = \frac{dP_{10}}{dt} = k_{22}P_9(P_{10tot} - P_{10}) - k_{23}P_{10}, \tag{119}$$

$$f_{11} = \frac{dP_{11}}{dt} = k_{24}P_6(P_{11tot} - P_{11}) - k_{25}P_{11}, \tag{120}$$

$$f_{12} = \frac{dP_{12}}{dt} = k_{26}(P_7 + k_{27}P_{11})(P_{12tot} - P_{12}) - k_{28}P_{12}, \tag{121}$$

$$f_{13} = \frac{dP_{13}}{dt} = k_{29}P_4(P_{13tot} - P_{13}) - k_{30}P_{13}, \tag{122}$$

$$f_{14} = \frac{dP_{14}}{dt} = k_{31}P_{13}(P_{14tot} - P_{14}) - k_{32}P_{14}, \tag{123}$$

$$f_{15} = \frac{dP_{15}}{dt} = k_{33}P_{14}(P_{15tot} - P_{15}) - k_{34}P_{15}P_{17}, \tag{124}$$

$$f_{16} = \frac{dP_{16}}{dt} = k_{35}P_{15}(P_{16tot} - P_{16}) - k_{36}P_{16}, \tag{125}$$

$$f_{17} = \frac{dP_{17}}{dt} = k_{37}P_{15} - k_{38}, \tag{126}$$

$$f_{18} = \frac{dP_{18}}{dt} = k_{39}(P_{12} + k_{40}P_{16})(P_{18tot} - P_{18}) - k_{41}P_{18}, \tag{127}$$

$$f_{19} = \frac{dP_{19}}{dt} = k_{42} - k_{43}P_5, \tag{128}$$

$$f_{20} = \frac{dP_{20}}{dt} = k_{44}(P_{20tot} - P_{20}) - k_{45}P_{20}P_{19}. \tag{129}$$

SN5.1.5 A Two-Node Opposing Set in a Network with a Shared Input/Output Node

As our final illustrative example, we demonstrate the implementation of RPA in a simple network in which the input node and the output node are one and the same node. We consider a representation of the network discussed by Ferrell [8] (which is itself a representation of the network proposed by [6]) as *antithetic integral feedback*, which we argue is an instance of a single Opposer Module that uses a two-node opposing set. Our representation of this network in Supplementary Figure 17, adapted from Figure 5 in [8] contains five nodes - A, B, C, D and P, where B is both the input node and the output node. As discussed elsewhere

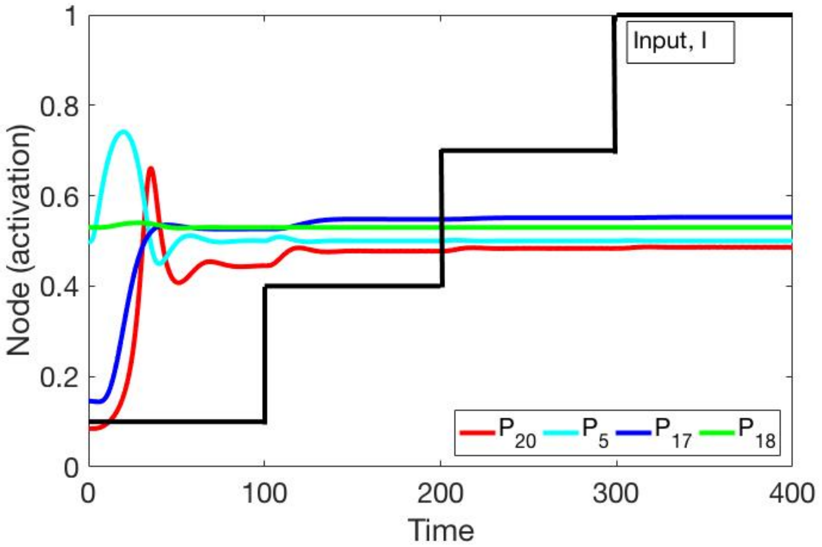

Supplementary Figure 16: Time-dependent solutions for modeling Equations (110) through (129). Nodes P_{17} and P_{19} act as opposer nodes, and thus need to operate with opposer kinetics (of which Equations (126) and (128) are the particular examples chosen here for illustrative purposes). The input stimulus, I, is represented in black and is a step function beginning at a level of 0.1, increasing in increments of 0.3 until a value of unity. As shown, the output node, P_{18} exhibits RPA, as does P_5 (which regulates the opposer P_{19}) and P_{15} (which regulates the opposer P_{17}, not shown). Nodes $P_6, P_7, P_8, P_9, P_{10}, P_{11}, P_{12}, P_{13}, P_{14}$ and P_{16} also exhibit RPA (not shown). Opposer nodes P_{17} and P_{19} are necessarily unable to exhibit RPA (P_{17} shown for illustrative purposes); Nodes $P_1, P_2, P_3, P_4, P_{13}, P_{14}$ as well as P_{20} (shown), are also unable to exhibit RPA for this particular solution. Parameters used in the modeling equations are: $k_1 = 1, k_2 = 0.2, k_3 = 1, k_4 = 1, k_5 = 0.2, k_6 = 1, k_7 = 0.2, k_8 = 1, k_9 = 0.2, k_{10} = 0.1, k_{11} = 0.1, k_{12} = 0.2, k_{13} = 0.4, k_{14} = 0.2, k_{15} = 0.4, k_{16} = 0.1, k_{17} = 0.2, k_{18} = 0.1, k_{19} = 0.2, k_{20} = 0.1, k_{21} = 0.2, k_{21a} = 0.1, k_{22} = 0.1, k_{23} = 0.2, k_{24} = 0.1, k_{25} = 0.2, k_{26} = 0.4, k_{27} = 0.1, k_{28} = 0.2, k_{29} = 0.2, k_{30} = 0.1, k_{31} = 0.2, k_{32} = 0.1, k_{33} = 0.1, k_{34} = 0.2, k_{35} = 0.2, k_{36} = 0.1, k_{37} = 0.2, k_{38} = 0.1, k_{39} = 0.4, k_{40} = 0.1, k_{41} = 0.2, k_{42} = 0.1, k_{43} = 0.2, k_{44} = 2, k_{45} = 20; P_{1tot} = P_{2tot} = P_{3tot} = P_{4tot} = P_{5tot} = P_{6tot} = P_{7tot} = P_{8tot} = P_{9tot} = P_{10tot} = P_{11tot} = P_{12tot} = P_{13tot} = P_{14tot} = P_{15tot} = P_{16tot} = P_{17tot} = P_{18tot} = P_{19tot} = P_{20tot} = 1$.

in this document, the RPA equation for a network with a single input/output node contains no routes, but rather contains the set of all (disjoint) cycle combinations that do not involve the input/output node. As a consequence, such networks only admit RPA solutions via an opposition mechanism. For this particular network, the terms of the RPA equation are represented by

$$(+)(+)\,(A)\,(C)\,(D)\,(P)$$
$$(-)(-)\,(A)\,(C)\,(D \rightarrow P)$$
$$(-)(-)\,(A)\,(D)\,(C \rightarrow P)$$

We see that nodes C, D and P all participate in a circuit that includes (is contiguous with) B (the input/output node), but also participate in at least one circuit that is disjoint from B. We see that the set $\{C, D\}$ meets the requirements of an opposing set of B, with an associated master set $\{C, D, P\}$. Thus, the network will exhibit RPA when both C and D operate with opposer kinetics. We use the same modeling equations as [8] (albeit adding an equation for the introduced node P in our representation of the network), namely

$$\frac{dA}{dt} = k_1 D - k_2 A, \tag{130}$$
$$\frac{dB}{dt} = k_e I A - k_4 B, \tag{131}$$
$$\frac{dC}{dt} = k_4 B - k_5 P, \tag{132}$$
$$\frac{dD}{dt} = k_6 - k_5 P, \tag{133}$$
$$\begin{aligned}\frac{dP}{dt} &= C\frac{dD}{dt} + D\frac{dC}{dt} \Leftrightarrow P = CD, \\ &= C(k_6 - k_5 P) + D(k_4 B - k_5 P).\end{aligned} \tag{134}$$

Time-course simulations of these network equations are presented in Supplementary Figure 18, using the parameter set given in the figure caption. Input, I, indicated in black is a step function beginning at 0.2 and increasing in increments of 0.2 until a value of 1. As expected, the output node, B exhibits RPA (as shown). Node P also adapts (not shown). The two opposer nodes, C and D, do not adapt as shown. Node A also does not adapt (not shown).

This particular example highlights the important point that a "node" is any quantity that plays a role in the encoding and transmission of biochemical signal through and around a network. Thus, while a node is *generally* a physical entity (ie. molecule - protein, RNA, protein activation state, etc) it could be a mathematical entity; in this specific example, the node P is the quantity CD.

SN5.2 Small Networks That Incorporate New Topological Features

We conclude this Supplementary Note with a consideration of how large network models need to be for computational searches to be able to discover RPA-capable topologies that use **both** the opposition mechanism **and** the balancing mechanism together to achieve RPA. We also consider the network size required for computational searching to identify the smallest versions of a (non-trivial) opposing set, and to identify the phenomenon of *feedforward opposition* - that is, opposer nodes that appear in feedback loops (as required) *as well as in a route*, thereby also playing a transmissive role within the network. Ma et al [15] had searched extensively on three-node networks, and were able to identify the simplest versions of the Opposer Module (which they referred to as *a negative feedback loop with buffering node*) and minimal versions of the Balancer Module (which they called *an incoherent feedforward loop with proportioner node*).

In Figure 8 of the main paper we present six small networks that are able to incorporate one or more of these novel topological features. Below, we provide a set of simple reaction equations for each of those those small networks; in Supplementary Figures 19 and 20 we present numerical simulations for those model equations (with specified parameters) to demonstrate that these particular network topologies can indeed engender RPA.

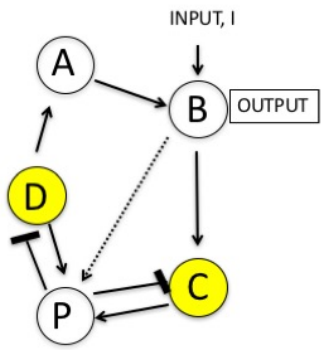

Supplementary Figure 17: Network schematic for a two-node feedback opposing set, with a single node (B) acting as both input node and output node. This example was presented and discussed in [8] as "antithetical integral feedback". The two-node opposing set, $\{C, D\}$ is indicated in yellow; the associated master set is $\{C, D, P, \}$

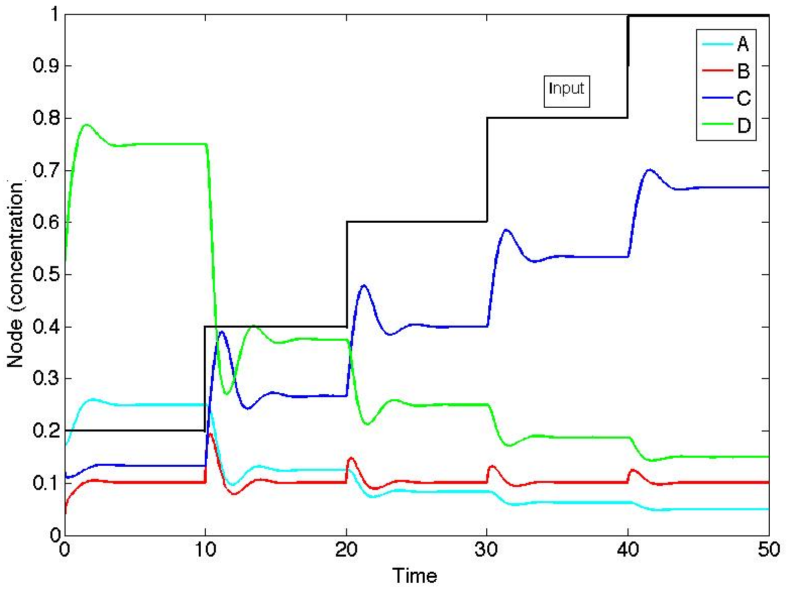

Supplementary Figure 18: Time-dependent solutions to the modeling equations 130 through 134. Parameters are: $k_1 = 1, k_2 = 3, k_3 = 20, k_4 = 10, k_5 = 10, k_6 = 1$.

Case a: Opposer module (upstream) connected in series with a balancer module (downstream)

$$
\begin{aligned}
f_I &= \frac{dI}{dt} = k_1(Input) - k_2 I - k_3 O_1,\\
f_O &= \frac{dO}{dt} = k_4 I + k_5 C - k_6 O,\\
f_{O_1} &= \frac{dO_1}{dt} = k_7 I - k_8,\\
f_C &= \frac{dC}{dt} = k_9 O_1 - k_{10} BC,\\
f_B &= \frac{dB}{dt} = k_{11} O_1 + k_{12} B.
\end{aligned}
$$

Case b: Balancer module (upstream) connected in series with an opposer module (downstream)

$$
\begin{aligned}
f_I &= k_1(Input) - k_2 I,\\
f_B &= k_3 I - k_4 B,\\
f_X &= \frac{k_5 B}{O_1} - k_7 X,\\
f_{O_1} &= k_8 X - k_9,\\
f_O &= k_{10} IX - k_{11} OB.
\end{aligned}
$$

Case c: Two opposer modules connected in series

$$
\begin{aligned}
f_I &= k_1(Input) - k_2 I - k_3 O_1,\\
f_O &= k_4 I + k_5 X - k_6 O,\\
f_{O_1} &= k_7 I - k_8,\\
f_X &= k_9 O_1 - k_{10} X - k_{11} O_2,\\
f_{O_2} &= k_{12} X - k_{13}.
\end{aligned}
$$

Case d: A single opposer module (employing a two-node opposing set)

$$
\begin{aligned}
f_I &= k_1(Input) - k_2 I + k_3 O_1,\\
f_O &= k_4 I - k_5 O,\\
f_{O_1} &= k_{11} X - k_{12},\\
f_X &= k_8 O_2 - k_9 X - k_{10} O_1,\\
f_{O_2} &= k_6 O - k_7 X.
\end{aligned}
$$

Case e: An opposer module (upstream, employing a two-node opposing set) connected in series with a balancer module (downstream)

$$
\begin{aligned}
f_I &= k_1(Input) - k_2 O_1 I,\\
f_{O_1} &= k_4 I - k_5 X,\\
f_X &= k_6 O_1 - k_7 O_2 - k_8 X,\\
f_{O_2} &= k_9 X - k_{10},\\
f_B &= k_{11} O_2 - k_{12} B,\\
f_C &= k_{14} O_2 - k_{15} BC,\\
f_O &= k_{16} I + k_{17} C - k_{18} O.
\end{aligned}
$$

Case f: An opposer module employing a two-node opposing set, connected in series with another opposer module (downstream, with single opposer node)

$$
\begin{aligned}
f_I &= k_1(Input) - k_2 I - k_3 O_1,\\
f_{O_1} &= k_4 I - k_5 X_1,\\
f_{X_1} &= k_6 O_1 - k_7 O_2 - k_8 X_1,\\
f_{O_2} &= k_9 X_1 - k_{10},\\
f_{X_2} &= k_{11} O_2 - k_{12} X_2 - k_{13} O_3,\\
f_{O_3} &= k_{14} X_2 - k_{15},\\
f_O &= k_{16} I + k_{17} X_2 - k_{18} O.
\end{aligned}
$$

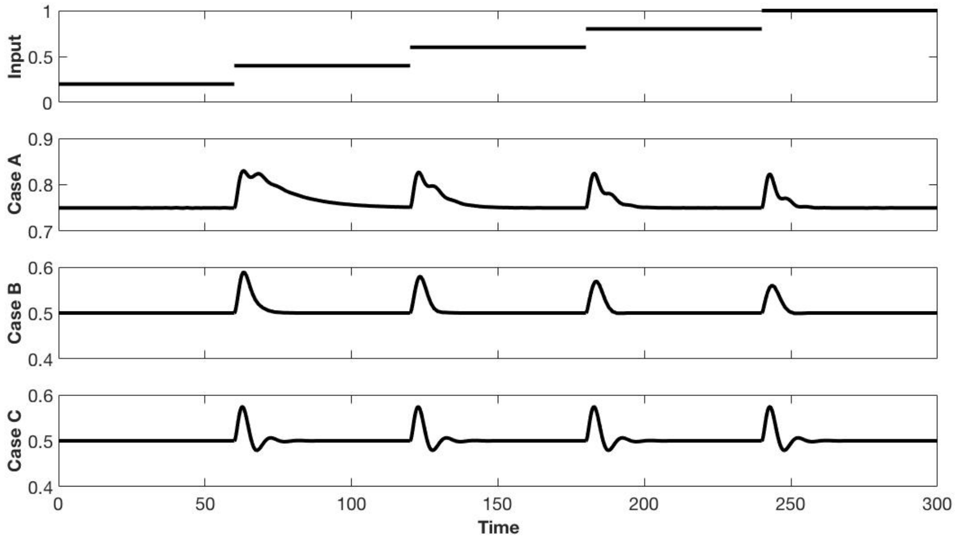

Supplementary Figure 19: Numerical simulations for Cases A, B and C depicted in Figure 8 of the main paper. Model equations for each case are listed in Section above. The activity level of the output node (O) is displayed in each case. The value of the Input is displayed in the top row: as shown, it increases from 0.2 to 1 in steps of 0.2. Parameter values used for these simulations are as follows: (a) $k_1 = k_3 = k_6 = k_7 = 1; k_2 = k_4 = k_5 = k_8 = k_9 = k_{10} = k_{11} = k_{12}$=0.5; (b) $k_1 = k_2 = k_3 = k_4 = k_7 = k_8 = k_{10} = k_{11} = 1; k_5 = k_9 = 0.5$. (c) $k_1 = k_3 = k_6 = k_7 = k_{12} = 1; k_2 = k_4 = k_5 = k_8 = k_9 = k_{10} = k_{11} = k_{13} = 0.5$.

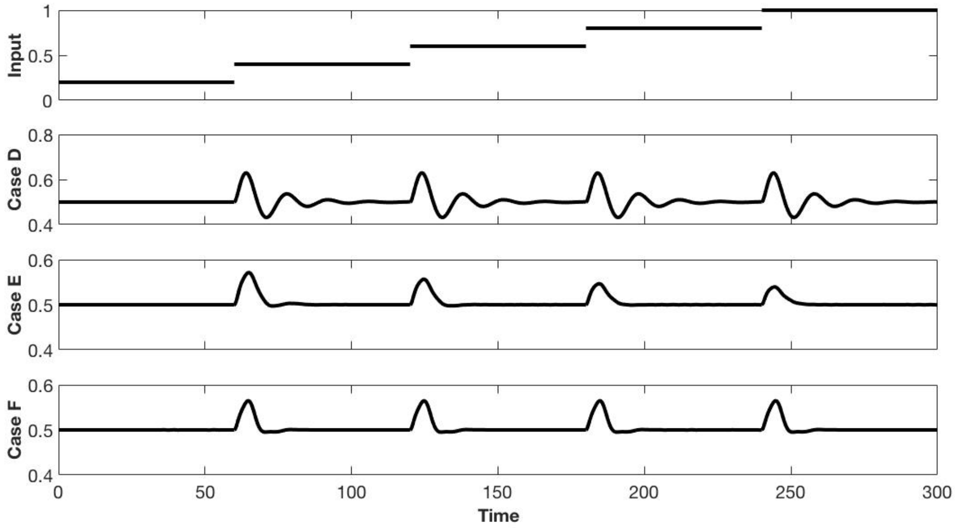

Supplementary Figure 20: Numerical simulations for Cases D, E and F depicted in Figure 8 of the main paper. Model equations for each case are listed in Section above. The activity level of the output node (O) is displayed in each case. The value of the Input is displayed in the top row: as shown, it increases from 0.2 to 1 in steps of 0.2. Parameter values used for these simulations are as follows: (d) $k_1 = k_2 = k_3 = k_9 = k_{12} = 0.5; k_4 = k_5 = k_6 = k_7 = k_8 = k_{10} = k_{11}$=1; (e) $k_2 = k_3 = k_4 = k_5 = k_6 = k_7 = k_9 = k_{11} = k_{12} = k_{13} = k_{15} = k_{18} = 1; k_1 = k_{10} = k_{14} = k_{16} = k_{17} = 0.5; k_8 = 0.4$. (f) $k_1 = k_2 = k_3 = k_{10} = k_{12} = k_{15} = k_{16} = k_{17} = 0.5; k_4 = k_5 = k_6 = k_7 = k_8 = k_9 = k_{11} = k_{13} = k_{14} = k_{18} = 1$.

Supplementary Note 6: Two Special Cases of RPA - Trivial Steady-States and "Local" (One-Node) RPA

As has been amply emphasized throughout this work, our aim is to provide a comprehensive and truly general understanding of robust perfect adaptation in biochemical networks – one that can accommodate arbitrarily large numbers of nodes and an arbitrarily high level of network complexity, one that is not restricted to trivial or otherwise "special" network steady-states, and one that escapes the need for special conditions such as particular choices of network parameters.

For the purpose of completeness, we note two such special instances of RPA - examples which, despite the special requirements of their implementation, have nevertheless been identified in certain biological contexts and have been shown to offer important functional utility in those particular applications. Importantly, both of these special cases are inherently local, being implemented at the level of a single network node. As such, RPA is not a network property in these cases.

The first of these arises from the special case where $\frac{\partial f_I}{\partial \mathscr{I}} = 0$ (at the network's steady-state), which implies reaction kinetics at the input node of the general form

$$f_I = g(\mathscr{I})h_1(P_u) - g(\mathscr{I})h_2(P_d), \tag{135}$$

where P_u and P_d represent the node(s) which upregulate and downregulate the P_I reaction (nodes which could include P_I itself), respectively. Thus, the input node both upregulates and downregulates the input node in the same way, representing the activity of a "paradoxical component" such as a bifunctional enzyme. Bifunctional enzymes, and their "paradoxical" effects on their targets have been reported to play a role in certain cases of signaling robustness (see, for example, [13], [14] and references therein).

We note, further, that the RPA-inducing effects of such a paradoxical component could be realised anywhere in a network, not just at the input node. If any node P_i acts as a bifunctional enzyme (say), as encoded by an equation of the form (135), on some target node P_j, then $\frac{\partial f_j}{\partial P_i} = 0$ for all $\mathscr{I}$ at the steady-state, which means that any route containing the link $\frac{\partial f_j}{\partial P_i}$ will be assigned to an S-set by the local (paradoxical) activity of P_j.

There is a sense in which these paradoxical regulations of a single node represent a trivial case of a balancer module. As such, the single node is essentially a connector node, whereas the upstream signal or regulation trivially plays the role of the balancer node(s).

The second special case concerns a recently identified phenomenon referred to as "state-dependent inactivation". This mechanism, too, occurs at the level of a single node. While this kind of mechanism has been reported for phenomena such as the activation and subsequent internalization of receptor tyrosine kinases (see [12] and [18] and references therein), the adaptive steady-state itself is constrained to be the trivial one - zero activity of the single target node. The special adaptive effects of this mechanism rely on the existence of an intermediate activation state of the molecule, and also rely on sufficient disparity between key reaction parameters; this allows a short-lived activation of the node before returning to the fully "off" state.

Supplementary Note 7: Correspondences between previously-identified perfect adaptation networks and the two RPA basis modules

Having shown that **all** networks capable of exhibiting RPA **must** be constructed from one or more of the five RPA basis modules, we remark that all previously identified cases of RPA are necessarily special cases of the general modular theory we have presented and discussed in this study.

In the Table below, we summarize the many previously reported biological examples of RPA (left-hand column) along with their known or putative RPA mechanism (right-hand column). In each case, the corresponding RPA module is highlighted in **bold**.

Supplementary Table 2: Previously-identified PA modules and their Relationships to RPA Basis Modules

BIOLOGICAL SYSTEM	RPA MODULE TYPE
Bacterial Chemotaxis [2, 4]	Integral Feedback Control - **Single Feedback Opposer Module**
Bacterial Nitrogen Assimilation [14]	Trivial Case of a **Balancer Module** (see Supp. Note 6)
Signal Transduction in mammalian cells - eg. EGFR-regulated signaling pathways [8, 17, 20]	Negative Feedback loops (with integral control) in the case of ERK-MKP1 - **Single Feedback Opposer Module**<br><br>Incoherent feedforward regulation in the case of EGFR-Ras signaling - **Balancer Module**
Yeast Osmoregulation [16]	Integral Feedback - **Single Feedback Opposer Module**
Regulation of Gene Expression - Prokaryotic transcription [6] and EGFR-regulated gene expression [8]	Antithetical Integral Feedback - **Two-node Feedback Opposing Set** - See our modeling simulations in SN5.1.5 of the example given by Ferrell [8]
Transcription Networks [11]	Incoherent Feedforward Control - **Balancer Module**
Calcium Homeostasis [7]	Integral Feedback - **Single Feedback Opposer Module**
Robustness and Scaling of Morphogen Gradients in Early Differentiation [3, 5]	Integral Feedback (application of the **Single Feedback Opposer Module** to a spatial signaling context)

Supplementary Note 8: A Note on Stability in RPA Networks

In this section we consider the conditions under which a perfectly-adaptive steady-state is a locally asymptotically stable one.

Now, to any n-node network we may associate a characteristic polynomial, π_n, given by

$$\pi_n = (-1)^n \det(\mathbf{J}_n - \lambda \mathbf{I}) = \lambda^n + a_1\lambda^{n-1} + a_2\lambda^{n-2} + \ldots + a_{n-1}\lambda + a_n, \qquad (136)$$

where, again, $\mathbf{J}_n$ is the system Jacobian matrix at the steady state, as presented in the earlier sections of this document, and λ represents the associated eigenvalues. To ensure (linear) stability of the steady state, we require all roots of π_n to lie strictly in the left half of the complex plane. Moreover, with solutions located strictly off the imaginary axis, such a steady state is *hyperbolic*; under these circumstances, the Hartman-Grobman theorem guarantees that the flow of the nonlinear network is topologically conjugate to that of the linearized network in some neighborhood of the steady-state [19].

By the Routh-Hurwitz theorem, all roots of π_n lie strictly in the left half of the complex plane if and only if the determinants of all n Hurwitz matrices are positive - a condition we refer to hereafter as the Routh-Hurwitz stability criterion [1]. The Hurwitz matrices are generated by the following pattern:

$$H_1 = \begin{bmatrix} a_1 \end{bmatrix},$$

$$H_2 = \begin{bmatrix} a_1 & 1 \\ a_3 & a_2 \end{bmatrix},$$

$$H_3 = \begin{bmatrix} a_1 & 1 & 0 \\ a_3 & a_2 & a_1 \\ a_5 & a_4 & a_3 \end{bmatrix},$$

$$H_n = \begin{bmatrix} a_1 & 1 & 0 & 0 & \ldots & 0 \\ a_3 & a_2 & a_1 & 1 & \ldots & 0 \\ a_5 & a_4 & a_3 & a_2 & \ldots & 0 \\ \vdots & \vdots & \vdots & \vdots & \vdots & \vdots \\ 0 & 0 & 0 & 0 & \ldots & a_n \end{bmatrix},$$

such that $a_m = 0$ if $m > n$.

A direct consequence of this criterion is that a necessary (but not sufficient) condition for the roots of the characteristic polynomial to lie in the left half of the complex plane is that the n coefficients $a_1, \ldots, a_n$ all be strictly positive. This being the case, we consider the condition $a_i > 0$ for all $i \in \{1, \ldots, n\}$ the *primary* (necessary) condition for network stability, and refer to these coefficients hereafter as the *stability coefficients*.

We thus begin by elucidating the nature of the stability coefficients $a_1, \ldots, a_n$ for a general n-node network. Now, from a consideration of the origin of these coefficients in the matrix determinant given in Equation (136) above, it readily follows that

$$a_m = \Sigma\left((-1)^z\, m\text{-node cycle products}\right) \qquad (137)$$

for the n-node network, with $1 \le m \le n$, and where z is the number of distinct cycles present in each term.

For $n = 2$, for instance, we see that

$$a_1 = -\frac{\partial f_1}{\partial P_1} - \frac{\partial f_2}{\partial P_2} = -\mathrm{Tr}(J_2),$$
$$a_2 = \frac{\partial f_1}{\partial P_1}\frac{\partial f_2}{\partial P_2} - \frac{\partial f_1}{\partial P_2}\frac{\partial f_2}{\partial P_1} = \det(J_2).$$

For $n = 3$,

$$\begin{aligned}
a_1 &= -\frac{\partial f_1}{\partial P_1} - \frac{\partial f_2}{\partial P_2} - \frac{\partial f_3}{\partial P_3} = -\mathrm{Tr}(J_3),\\
a_2 &= \frac{\partial f_1}{\partial P_1}\frac{\partial f_2}{\partial P_2} + \frac{\partial f_1}{\partial P_1}\frac{\partial f_3}{\partial P_3} + \frac{\partial f_2}{\partial P_2}\frac{\partial f_3}{\partial P_3} - \frac{\partial f_1}{\partial P_2}\frac{\partial f_2}{\partial P_1} - \frac{\partial f_1}{\partial P_3}\frac{\partial f_3}{\partial P_1} - \frac{\partial f_2}{\partial P_3}\frac{\partial f_3}{\partial P_2},\\
a_3 &= -\frac{\partial f_1}{\partial P_1}\frac{\partial f_2}{\partial P_2}\frac{\partial f_3}{\partial P_3} + \frac{\partial f_1}{\partial P_1}\frac{\partial f_2}{\partial P_3}\frac{\partial f_3}{\partial P_2} - \frac{\partial f_1}{\partial P_2}\frac{\partial f_2}{\partial P_3}\frac{\partial f_3}{\partial P_1} + \frac{\partial f_1}{\partial P_2}\frac{\partial f_2}{\partial P_1}\frac{\partial f_3}{\partial P_3} - \frac{\partial f_1}{\partial P_3}\frac{\partial f_3}{\partial P_2}\frac{\partial f_2}{\partial P_1} + \frac{\partial f_1}{\partial P_3}\frac{\partial f_3}{\partial P_1}\frac{\partial f_2}{\partial P_2}\\
&= -\det(J_3).
\end{aligned}$$

It should be noted, of course, that for a *particular* network, circuits only appear in the expansions for the a_i coefficients if those circuits actually exist in the network in question. Moreover, since the maximum number of terms in a_n for an n-node network is $n!$, we see that these coefficient expansions grow factorially (ie. extremely rapidly) with network size.

For case (a) in Supplementary Figure 21, for example, taking the node P_2 to be an opposer node to form a single opposer module, the stability coefficients are

$$\begin{aligned}
a_1 &= -\frac{\partial f_1}{\partial P_1} - \frac{\partial f_3}{\partial P_3},\\
a_2 &= \frac{\partial f_1}{\partial P_1}\frac{\partial f_3}{\partial P_3},\\
a_3 &= -\frac{\partial f_1}{\partial P_2}\frac{\partial f_3}{\partial P_1}\frac{\partial f_2}{\partial P_3},
\end{aligned}$$

from which it follows that the primary stability condition is satisfied with negative kinetic multipliers for the non-opposer nodes P_1 and P_3, and the negative three-node feedback loop indicated in the diagram. To guarantee (local) stability, however, the three Hurwitz matrices must have positive determinants. In this case, $\det(H_1)=a_1$, $\det(H_2)=a_1 a_2 - a_3$, and $\det(H_3)=\det(H_2) a_3$. Thus, in addition to positive stability coefficients, we additionally require $a_1 a_2 > a_3$, ie.$-\frac{\partial f_1}{\partial P_1}\frac{\partial f_1}{\partial P_1}\frac{\partial f_3}{\partial P_3} - \frac{\partial f_3}{\partial P_3}\frac{\partial f_1}{\partial P_1}\frac{\partial f_1}{\partial P_1} > -\frac{\partial f_1}{\partial P_2}\frac{\partial f_3}{\partial P_1}\frac{\partial f_2}{\partial P_3}$. Thus, it is possible for such a network configuration to fail to meet all Routh-Hurwitz criteria (specifically, $\det(H_2)$) in some parameter regimes.

For case (b) in Supplementary Figure 21, on the other hand, taking the node P_2 to be a balancer node, and node P_3 to be a connector node, forming a simple balancer module, the stability coefficients are

$$\begin{aligned}
a_1 &= -\frac{\partial f_1}{\partial P_1} - \frac{\partial f_2}{\partial P_2} - \frac{\partial f_3}{\partial P_3},\\
a_2 &= \frac{\partial f_1}{\partial P_1}\frac{\partial f_2}{\partial P_2} + \frac{\partial f_1}{\partial P_1}\frac{\partial f_3}{\partial P_3} + \frac{\partial f_2}{\partial P_2}\frac{\partial f_3}{\partial P_3},\\
a_3 &= -\frac{\partial f_1}{\partial P_1}\frac{\partial f_2}{\partial P_2}\frac{\partial f_3}{\partial P_3}.
\end{aligned}$$

Once again, with negative kinetic multipliers, the primary stability condition is safisfied. Now, the Routh-Hurwitz conditions additionally require $a_1 a_2 > a_3$, which in this case corresponds to

$$\begin{aligned}
&-\frac{\partial f_1}{\partial P_1}\frac{\partial f_1}{\partial P_1}\frac{\partial f_2}{\partial P_2} - \frac{\partial f_1}{\partial P_1}\frac{\partial f_1}{\partial P_1}\frac{\partial f_3}{\partial P_3} - \frac{\partial f_1}{\partial P_1}\frac{\partial f_2}{\partial P_2}\frac{\partial f_3}{\partial P_3} - \frac{\partial f_2}{\partial P_2}\frac{\partial f_1}{\partial P_1}\frac{\partial f_2}{\partial P_2} - \frac{\partial f_2}{\partial P_2}\frac{\partial f_1}{\partial P_1}\frac{\partial f_3}{\partial P_3}\\
&-\frac{\partial f_2}{\partial P_2}\frac{\partial f_2}{\partial P_2}\frac{\partial f_3}{\partial P_3} - \frac{\partial f_3}{\partial P_3}\frac{\partial f_1}{\partial P_1}\frac{\partial f_2}{\partial P_2} - \frac{\partial f_3}{\partial P_3}\frac{\partial f_1}{\partial P_1}\frac{\partial f_3}{\partial P_3} - \frac{\partial f_3}{\partial P_3}\frac{\partial f_2}{\partial P_2}\frac{\partial f_3}{\partial P_3}\\
&= -3\left(\frac{\partial f_1}{\partial P_1}\frac{\partial f_2}{\partial P_2}\frac{\partial f_3}{\partial P_3}\right) - \frac{\partial f_1}{\partial P_1}\frac{\partial f_1}{\partial P_1}\frac{\partial f_2}{\partial P_2} - \frac{\partial f_1}{\partial P_1}\frac{\partial f_1}{\partial P_1}\frac{\partial f_3}{\partial P_3} - \frac{\partial f_2}{\partial P_2}\frac{\partial f_1}{\partial P_1}\frac{\partial f_2}{\partial P_2}\\
&-\frac{\partial f_2}{\partial P_2}\frac{\partial f_2}{\partial P_2}\frac{\partial f_3}{\partial P_3} - \frac{\partial f_3}{\partial P_3}\frac{\partial f_1}{\partial P_1}\frac{\partial f_3}{\partial P_3} - \frac{\partial f_3}{\partial P_3}\frac{\partial f_2}{\partial P_2}\frac{\partial f_3}{\partial P_3}\\
&> -\frac{\partial f_1}{\partial P_1}\frac{\partial f_2}{\partial P_2}\frac{\partial f_3}{\partial P_3},
\end{aligned}$$

which is automatically satisfied for negative kinetic multipliers (which produce positive stability coefficients) for this specific network configuration.

We note that while the primary stability conditions will be met for *any* network with purely negative cycles, the Routh-Hurwitz conditions that guarantee stability become increasingly complex with increasing network size (number of nodes), especially for networks involving many circuits (feedback loops), since the stability coefficients that generate the Hurwitz matrices and their determinants are composed of all combinations of the cycle products in a network. For case (c) in Supplementary Figure 21, for instance, taking the node P_2 to be an opposer node to create single opposer module from the five-node arrangement, the stability coefficients are

$$
\begin{aligned}
a_1 &= -\frac{\partial f_1}{\partial P_1} - \frac{\partial f_3}{\partial P_3} - \frac{\partial f_4}{\partial P_4} - \frac{\partial f_5}{\partial P_5}, \\
a_2 &= \frac{\partial f_1}{\partial P_1}\frac{\partial f_3}{\partial P_3} + \frac{\partial f_1}{\partial P_1}\frac{\partial f_4}{\partial P_4} + \frac{\partial f_1}{\partial P_1}\frac{\partial f_5}{\partial P_5} + \frac{\partial f_3}{\partial P_3}\frac{\partial f_4}{\partial P_4} + \frac{\partial f_3}{\partial P_3}\frac{\partial f_5}{\partial P_5} + \frac{\partial f_4}{\partial P_4}\frac{\partial f_5}{\partial P_5}, \\
a_3 &= -\frac{\partial f_1}{\partial P_1}\frac{\partial f_3}{\partial P_3}\frac{\partial f_4}{\partial P_4} - \frac{\partial f_1}{\partial P_1}\frac{\partial f_3}{\partial P_3}\frac{\partial f_5}{\partial P_5} - \frac{\partial f_1}{\partial P_1}\frac{\partial f_4}{\partial P_4}\frac{\partial f_5}{\partial P_5} - \frac{\partial f_3}{\partial P_3}\frac{\partial f_4}{\partial P_4}\frac{\partial f_5}{\partial P_5}, \\
a_4 &= \frac{\partial f_1}{\partial P_1}\frac{\partial f_3}{\partial P_3}\frac{\partial f_4}{\partial P_4}\frac{\partial f_5}{\partial P_5}, \\
a_5 &= \frac{\partial f_1}{\partial P_2}\frac{\partial f_2}{\partial P_5}\frac{\partial f_5}{\partial P_4}\frac{\partial f_4}{\partial P_3}\frac{\partial f_3}{\partial P_1},
\end{aligned}
$$

which, once again, are guaranteed to be positive (as required) if all kinetic multipliers are negative with the five-node circuit negative (as depicted). An analysis of the five Hurwitz matrices shows that the following additional two criteria must be satisfied in order to guarantee stability -

$$
\begin{gathered}
a_1 a_2 a_3 > {a_3}^2 + {a_1}^2 a_4, \\
(a_1 a_4 - a_5)(a_1 a_2 a_3 - {a_3}^2 - {a_1}^2 a_4) > a_5 (a_1 a_2 - a_3)^2 a_1 {a_5}^2.
\end{gathered}
$$

underscoring the fact that the difficulty of making a definite determination as to stability increases rapidly with network size (number of nodes) and complexity (which in the context of stability refers to the presence of more feedback circuits in the network).

Moreover, even when a steady-state is locally asymptotically stable, this is no guarantee that the steady-state is globally asymptotically stable, as in principle, a network may possess multiple steady states, and the system could be stimulated from away from the adaptive set-point to an alternative steady-state under certain circumstances.

The main conclusion to be drawn, for the purposes of the present work, is that *negative* cycles (ie. negative kinetic multipliers and negative feedback loops, in the sense of an odd number of inhibitory interactions within the circuit) promote local asymptotic stability of the steady-state for an RPA solution. Indeed, having *only* negative cycles guarantees that the *primary* stability conditions will be met. As such, we consider negative cycles to be the generic property of RPA networks. Indeed, all specific RPA networks analyzed in this work for illustrative purposes (Supplementary Note 5) were assigned purely negative cycles. In each case time-dependent solutions were produced, and stable RPA solutions were readily obtained, thus supporting the notion that the presence of *only* negative cycles in an RPA network is conducive to (if not a guarantee of) a stable (and thus 'true') RPA solution.

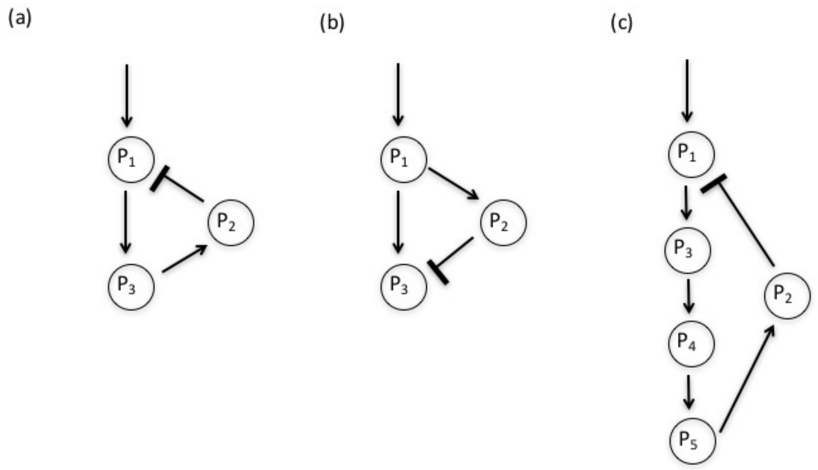

Supplementary Figure 21: Three simple network configurations, whose stability properties are examined in Supplementary Note 8.

Supplementary References

[1] Linda JS Allen. *Introduction to mathematical biology*. Pearson/Prentice Hall, 2007.

[2] Uri Alon, Michael G Surette, Naama Barkai, and Stanislas Leibler. Robustness in bacterial chemotaxis. *Nature*, 397(6715):168–171, 1999.

[3] Inna Averbukh, Danny Ben-Zvi, Siddhartha Mishra, and Naama Barkai. Scaling morphogen gradients during tissue growth by a cell division rule. *Development*, 141(10):2150–2156, 2014.

[4] Naama Barkai and Stan Leibler. Robustness in simple biochemical networks. *Nature*, 387(6636):913–917, 1997.

[5] Danny Ben-Zvi and Naama Barkai. Scaling of morphogen gradients by an expansion-repression integral feedback control. *Proceedings of the National Academy of Sciences*, 107(15):6924–6929, 2010.

[6] Corentin Briat, Ankit Gupta, and Mustafa Khammash. Antithetic integral feedback ensures robust perfect adaptation in noisy bimolecular networks. *Cell systems*, 2(1):15–26, 2016.

[7] H El-Samad, JP Goff, and M Khammash. Calcium homeostasis and parturient hypocalcemia: an integral feedback perspective. *Journal of Theoretical Biology*, 214(1):17–29, 2002.

[8] James E Ferrell. Perfect and near-perfect adaptation in cell signaling. *Cell systems*, 2(2):62–67, 2016.

[9] Bruce A Francis and W Murray Wonham. The internal model principle of control theory. *Automatica*, 12(5):457–465, 1976.

[10] Bruce A Francis and William M Wonham. The internal model principle for linear multivariable regulators. *Applied mathematics and optimization*, 2(2):170–194, 1975.

[11] Lea Goentoro, Oren Shoval, Marc W Kirschner, and Uri Alon. The incoherent feedforward loop can provide fold-change detection in gene regulation. *Molecular cell*, 36(5):894–899, 2009.

[12] Lai Kuan Goh and Alexander Sorkin. Endocytosis of receptor tyrosine kinases. *Cold Spring Harbor perspectives in biology*, 5(5):a017459, 2013.

[13] Yuval Hart and Uri Alon. The utility of paradoxical components in biological circuits. *Molecular cell*, 49(2):213–221, 2013.

[14] Yuval Hart, Daniel Madar, Jie Yuan, Anat Bren, Avraham E Mayo, Joshua D Rabinowitz, and Uri Alon. Robust control of nitrogen assimilation by a bifunctional enzyme in e. coli. *Molecular cell*, 41(1):117–127, 2011.

[15] Wenzhe Ma, Ala Trusina, Hana El-Samad, Wendell A Lim, and Chao Tang. Defining network topologies that can achieve biochemical adaptation. *Cell*, 138(4):760–773, 2009.

[16] Dale Muzzey, Carlos A Gómez-Uribe, Jerome T Mettetal, and Alexander van Oudenaarden. A systems-level analysis of perfect adaptation in yeast osmoregulation. *Cell*, 138(1):160–171, 2009.

[17] Satoru Sasagawa, Yu-ichi Ozaki, Kazuhiro Fujita, and Shinya Kuroda. Prediction and validation of the distinct dynamics of transient and sustained erk activation. *Nature cell biology*, 7(4):365–373, 2005.

[18] Alexander Sorkin and Lai Kuan Goh. Endocytosis and intracellular trafficking of erbbs. *Experimental cell research*, 315(4):683–696, 2009.

[19] Steven H Strogatz. *Nonlinear dynamics and chaos: with applications to physics, biology, chemistry, and engineering*. Westview press, 2014.

[20] Hong Sun, Catherine H Charles, Lester F Lau, and Nicholas K Tonks. Mkp-1 (3ch134), an immediate early gene product, is a dual specificity phosphatase that dephosphorylates map kinase in vivo. *Cell*, 75(3):487–493, 1993.

[21] Tau-Mu Yi, Yun Huang, Melvin I Simon, and John Doyle. Robust perfect adaptation in bacterial chemotaxis through integral feedback control. *Proceedings of the National Academy of Sciences*, 97(9):4649–4653, 2000.
